# Supplementary material for: The most energetic transients: Tidal disruptions of high-mass stars
Source: Sci Adv. 2025 Jun 4;11(23):eadt0074. doi: 10.1126/sciadv.adt0074 (PMC13109975; doi:10.1126/sciadv.adt0074)
Supplement: Supplementary file 1 — Supplementary Text Figs. S1 to S8 Tables S1 to S3 References [file sciadv.adt0074_sm.pdf]

Science Advances
AAAS

Supplementary Materials for

The most energetic transients: Tidal disruptions of high-mass stars

Jason T. Hinkle *et al.*

Corresponding author: Jason T. Hinkle, jhinkle6@hawaii.edu

Sci. Adv. **11**, eadt0074 (2025)
DOI: 10.1126/sciadv.adt0074

This PDF file includes:

Supplementary Text
Figs. S1 to S8
Tables S1 to S3
References

Observational Data

Sample Selection When implementing our search criteria we supplemented the Gaia photometry with archival photometry from the All-Sky Automated Survey for Supernovae (ASAS-SN; (*3, 179, 180*)), Asteroid Terrestrial-impact Last Alert System (ATLAS; (*26*)), the Catalina Real-Time Transient Survey (CRTS; (*62*)), and the Zwicky Transient Facility (ZTF; (*181,182*)). We also obtained optical spectra with the UH 2.2-m telescope and the Spectral Classification of Astronomical Transients (SCAT; (*183*)) to vet several other smooth nuclear transients which were ultimately rejected because their redshifts indicated lower peak luminosities not sufficient to meet criterion (4). We finally used the Fermi Large Area Telescope Catalog (*184*) to search for gamma-ray detections and Vizier (*185*) to search several radio catalogs across the sky.

The three transients we study in detail are Gaia16aaw, Gaia18cdj, and ZTF20abrbeie. Gaia16aaw, (α, δ) = (04:11:57.000, −42:05:30.80), was discovered on 2016 January 23.5 by the Gaia Alerts team (*27*). The discovery was announced publicly on the Transient Name Server (TNS) and given the identification AT2016dbs (*186*). Gaia18cdj, (α, δ) = (02:09:48.140, −42:04:37.02), was discovered on 2018 August 12.1 by the Gaia Alerts team (*27*) and announced on TNS with the identification AT2018fbb (*187*). ZTF20abrbeie, (α, δ) = (21:13:48.408, +27:25:50.48), was discovered on 2021 April 13.5 by ZTF. It was announced to TNS with the identification AT2021lwx (*188*). Color images created using APLPY (*189*) from gri images taken by either the Dark Energy Survey (DES; for Gaia16aaw and Gaia18cdj) (*64*) or the Panoramic Survey Telescope and Rapid Response System (Pan-STARRS; for AT2021lwx) (*190*) are shown in figure S1.

As ZTF20abrbeie has had previous papers published on its evolution under the name AT2021lwx (*29, 30*), we elect to use that name in this manuscript to avoid confusion. For the two Gaia sources, we elect to use their survey names in this manuscript.

Archival and Transient Photometry We first searched for available archival photometry to constrain the evolution of these objects prior to the ENT flare. For Gaia16aaw and Gaia18cdj, we obtained V-band photometry from the CRTS. As the typical signal-to-noise ratio (S/N) per CRTS epoch was low, we binned these data in monthly bins to search for pre-flare variability. These data are shown in Figure 1 as tan squares. The sources are weakly detected and show no significant evidence for strong variability prior to the flares as any pre-flare variability in CRTS data for Gaia16aaw and Gaia18cdj is consistent with noise.

To constrain both the pre-flare characteristics of the objects and their behavior during the flare, we also obtained photometry from the Wide-field Infrared Survey Explorer (WISE; (*63*)). This includes data taken during both the AllWISE (*63*) and NEOWISE (*191, 192*) portions of the WISE mission. We have focused on the $W1$ (3.4 μm) and $W2$ (4.6 μm) bands as they span the full lifetime of the WISE mission. Gaia16aaw and Gaia18cdj are well-detected in the NEOWISE single exposure catalog, and we therefore construct their $W1$ and $W2$ light curves by binning the individual exposures within a given epoch.

For AT2021lwx, there is no persistent source detected in the NEOWISE single exposure catalog, consistent with the apparent lack of a host galaxy in Pan-STARRS imaging (*29, 30*). We therefore performed aperture photometry on the NEOWISE images to obtain our WISE light curves. To avoid contamination by nearby, bright stars, we used a 4” radius aperture with an aperture correction estimated from the computed WISE curve of growth. The local background was estimated using a sigma-clipped median within an annulus around the target. Despite the small source aperture, the $W1$ band retained a low level of residual flux from the nearby, bright star. To mitigate this, we subtracted the mean pre-flare flux from the light curves and added the pre-flare scatter in quadrature with the estimated flux uncertainties for each epoch. The WISE light curves are shown in Figure 1 as olive and brown diamonds for $W1$ and $W2$ respectively. Given the lack of a pre-flare detection, we only show NEOWISE data for AT2021lwx.

DES (*64*) imaging covered the locations of Gaia16aaw and Gaia18cdj. These images span from late 2013 to late 2018 and cover these locations both prior to and during the ENT. We created DES light curves in the $griz$ bands by performing aperture photometry with a 3" radius aperture, subtracting the local background estimated through a sigma-clipped median in an annulus centered on the source, and calibrating to nearby stars with catalog photometry from ATLAS Refcat2 (*193*). We elected to use a relatively large aperture to ensure that the entire galaxy was contained within the aperture. The DES data are shown in Figure 1 as circles, with the color corresponding to the filter.

For each ENT, we also obtained ATLAS light curves from their forced point-spread function photometry service. This yielded light curves in the 'cyan' (c, 4200–6500 Å) and 'orange' (o, 5600–8200 Å) filters (*26*). To ensure more robust detections, we stacked the ATLAS data in monthly bins throughout the flares. The ATLAS photometry is plotted in Figure 1 as cyan and orange pentagons.

Each of the sources was observed by the Neil Gehrels Swift Gamma-ray Burst Mission (*Swift*; (*65*)) at least once during its evolution. These observations utilized both the UltraViolet and Optical Telescope (UVOT; (*194*)) and X-ray Telescope (XRT; (*195*)). We combined all UVOT images taken in a given filter per epoch using the HEASoft `uvotimsum` package and used the `uvotsource` package to extract source and background fluxes from these coadded images. For Gaia16aaw (PI: Hinkle) and Gaia18cdj (PI: Hinkle) we used the default UVOT aperture with a radius of 5" and background regions with radii of 50" and 40", respectively. For AT2021lwx (PI: Wang), there is a star of comparable brightness within the default 5" aperture and so we used a smaller 3" aperture to capture only the source flux. As AT2021lwx is relatively close to the Galactic plane and is therefore in a moderately crowded field, we used a background ellipse with an effective area of ≈27" chosen to avoid contamination from nearby stars.

Finally, we acquired light curves from the discovery survey for each source. For Gaia16aaw

and Gaia18cdj, this was G-band photometry from Gaia, obtained through the Gaia Alerts service. We estimated the uncertainties on this photometry following (*196*) and binned visits within 5 days for subsequent analyses. The Gaia light curves are shown in Figure 1 as green hexagons. For AT2021lwx, we procured ZTF photometry in the g and r bands from the ZTF forced photometry service (*182*). We stacked the pre-flare data in monthly bins to obtain deep limits. During the rise and through peak, we stacked the data in 5-day bins, switching to monthly bins after the last seasonal break to better sample the decline. These data are shown in Figure 1 as teal and red octagons.

Host-Galaxy Properties

Stellar Mass and Star Formation Rate We further examine the SFRs of these ENT host galaxies using narrow emission lines and standard scaling relations. Gaia16aaw shows an emission feature near [O II], although there is contamination from night sky lines. Assuming that this line is indeed [O II], we derive a flux of 6.1×10^{-17} erg s^{-1} cm^{-2}. At the distance of Gaia16aaw and correcting for the $A_V = 0.92$ mag extinction from the CIGALE fits, this corresponds to a luminosity of 1.3×10^{42} erg s^{-1}. From typical scaling relations (*197, 198*), such a luminosity implies a SFR of $\sim$ 10–20 M$_\odot$ yr^{-1}, roughly 2σ below the estimate from the CIGALE fits.

Gaia18cdj does not show an [O II] emission line, so we calculated an upper-limit on the [O II] luminosity using Equation (1) with a line width of 300 km s^{-1}. Correcting for the extinction from the CIGALE fits, this yields a luminosity upper-limit of $< 4 \times 10^{41}$ erg s^{-1}, implying an SFR of $<$ 3–6 M$_\odot$ yr^{-1} (*197, 198*). While the SFRs estimated from spectroscopic scaling relations are low relative to the estimates from CIGALE, we note (1) that these lines are in regions of the spectra with S/N below 5 and (2) that the SFRs estimated from the rest-frame UV absolute magnitudes of $M \approx -22.5$ mag (*197*) are consistent with the CIGALE estimates.

Properties of the Flares

Dust Covering Fraction Anisotropies in the dust distribution can cause the ratio of IR to UV/optical luminosity to underestimate low covering factors and overestimate high covering factors. While the emitting geometry of these ENTs is unknown, we apply the corrections of (*85*) to examine their effects. Using Table 1 of (*85*) and assuming an aligned disk and torus with $\tau_{9.7\mu m} = 3$, their minimum computed optical depth, we find covering fractions of $f_c \geq 0.42$, $f_c = 0.44$, and $f_c = 0.58$ for Gaia16aaw, Gaia18cdj, and AT2021lwx respectively. While these corrections increase the covering fraction estimates for our ENTs, their position relative to the TDE and ANT samples and the typical AGN trend in Fig. 8 are not dramatically different.

We also used JAVELIN (*199, 200*) to compute temporal lags between the optical and IR flares, employing a top hat smoothing function. For AT2021lwx, as the source is undetected pre-flare, we added artificial optical measurements at the time of the NEOWISE observations with zero flux and uncertainties equal to the first optical flux uncertainty. This was necessary to avoid significantly negative fluxes in the model optical light curve at early times. These fits are shown in figure S5. We find rest-frame lags of ≈265 days for Gaia16aaw, ≈150 days for Gaia18cdj, and ≈70 days for AT2021lwx, where these are the weighted average of the lags between the $W1$ and $W2$ bands and the optical band. The $W2$ lags are generally longer than the $W1$ lags, as we would expect, since the $W2$ emission will include contributions from cooler, more distant dust. The widths of the smoothing top hats were roughly twice the mean lag, which is the lag distribution of a spherical shell. This suggests that the dust subtends a large solid angle around the transient.

We can alternatively estimate the dust covering fractions as $f_c \approx (L_{IR}\Delta t)/(\tau_{dust}E_{flare})$ where L_{IR} is the peak IR luminosity of the dust echo,$\Delta\ t$ is the temporal lag, τ is the dust optical depth, and E_{flare} is the emitted energy of the transient. Assuming $\tau_{dust} = 0.25$, we find

dust covering fractions of $\sim$0.7 for Gaia16aaw, $\sim$0.2 for Gaia18cdj, and $\sim$0.3 for AT2021lwx, each in good agreement with the estimates from the luminosity ratios.

While it is not exact and depends on the dust composition in detail, the fraction of the observed optical light that consists of photons scattered by the dust should be comparable to the absorbed fraction. Hence, another argument that the overall fraction of the absorbed light must be modest is that if the mean optical depth of the dust producing the IR echoes is high, scattered photons from the peak UV/optical emission would overproduce the tail of the light curve. As it is, some fraction of the tail is likely from photons scattered off the dust. This should be a general consideration for models of mid-IR dust echoes from nuclear transients (e.g., (*201*)), although the detailed modeling of the dust radiative transfer is beyond the scope of this paper.

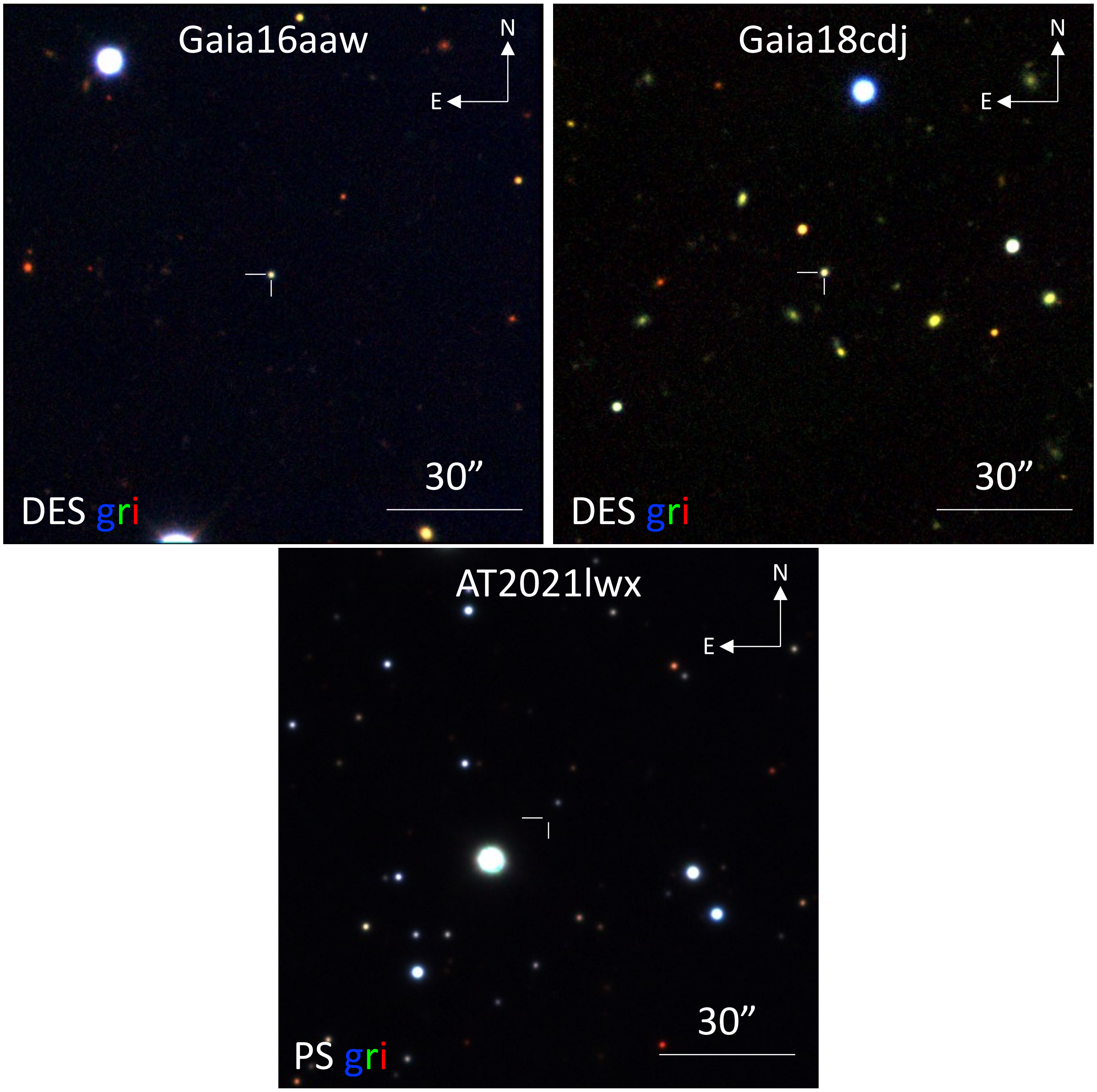

Figure S1: **Pre-flare *gri* color images Gaia16aaw (top left; DES), Gaia18cdj (top right; DES), and AT2011lwx (bottom; Pan-STARRS).** The white reticle in each image marks the location of the transient.

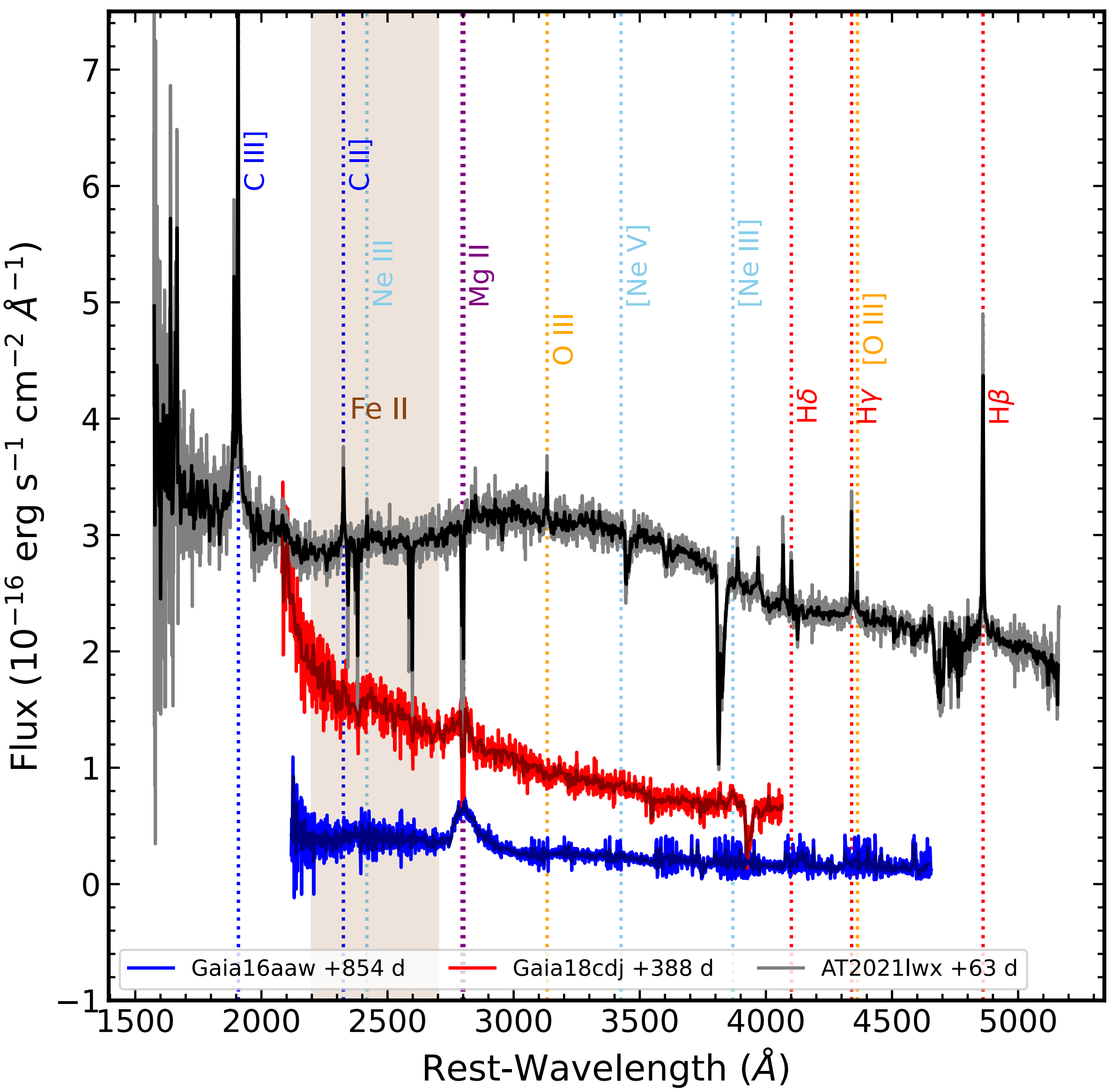

Figure S2: **Spectra of Gaia16aaw (blue), Gaia18cdj (red), and AT2021lwx (gray).** At the redshift of these sources, the observer-frame optical covers the rest-frame UV and blue portion of the optical. Marked are emission lines of C (blue), Ne (sky blue), Mg (purple), O (orange), H (red), and the broad feature due to Fe lines (brown). The legend indicates the time of observation in rest-frame days from peak.

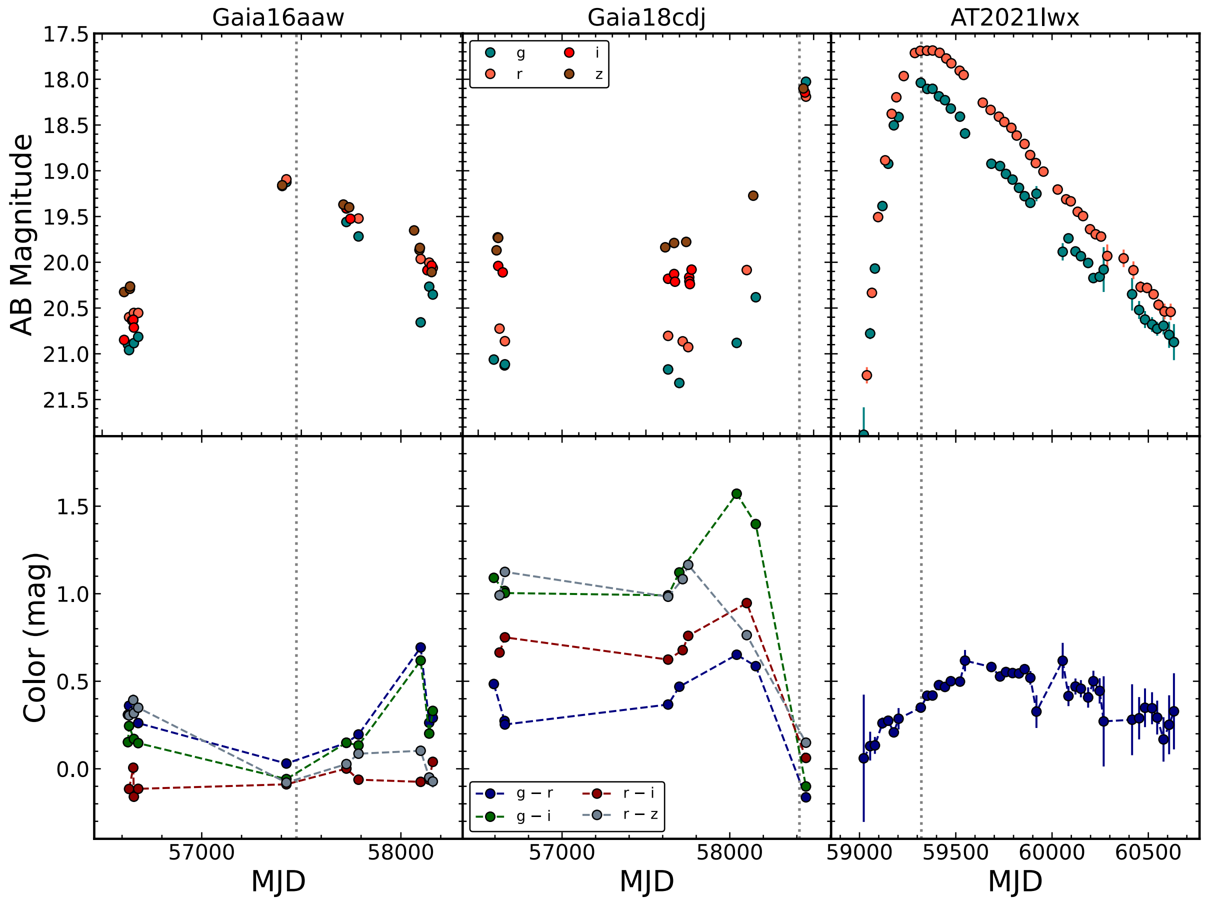

Figure S3: **Observed-frame optical light curves (top panel) and colors (bottom panel) for Gaia16aaw (left), Gaia18cdj (middle), and AT2021lwx (right).** For Gaia16aaw and Gaia18cdj, the data is from DES and for AT2021lwx the data is from ZTF. These light curves are corrected for Galactic foreground extinction, but have not had any host flux or host extinction removed. The dashed vertical gray line marks the time of peak for each source.

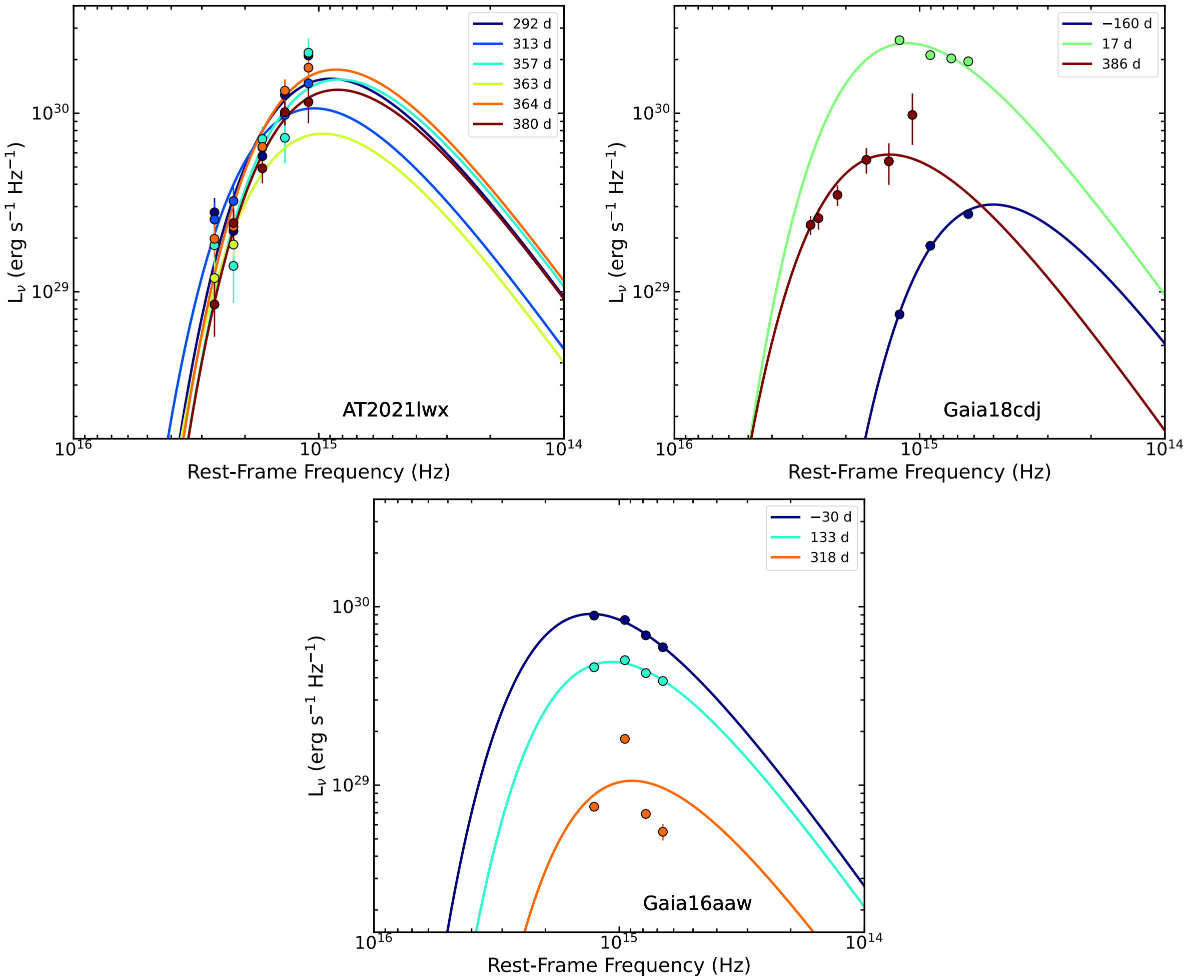

Figure S4: **Rest-frame UV/optical photometry and corresponding best-fitting blackbody models for AT2021lwx (top left), Gaia18cdj (top right), and Gaia16aaw (bottom).** For AT2021lwx, the data fit is from Swift UVOT, for Gaia18cdj the data comes from Swift and DES, and the data for Gaia16aaw is from DES. The times given in the legend are rest-frame phases from peak emission.

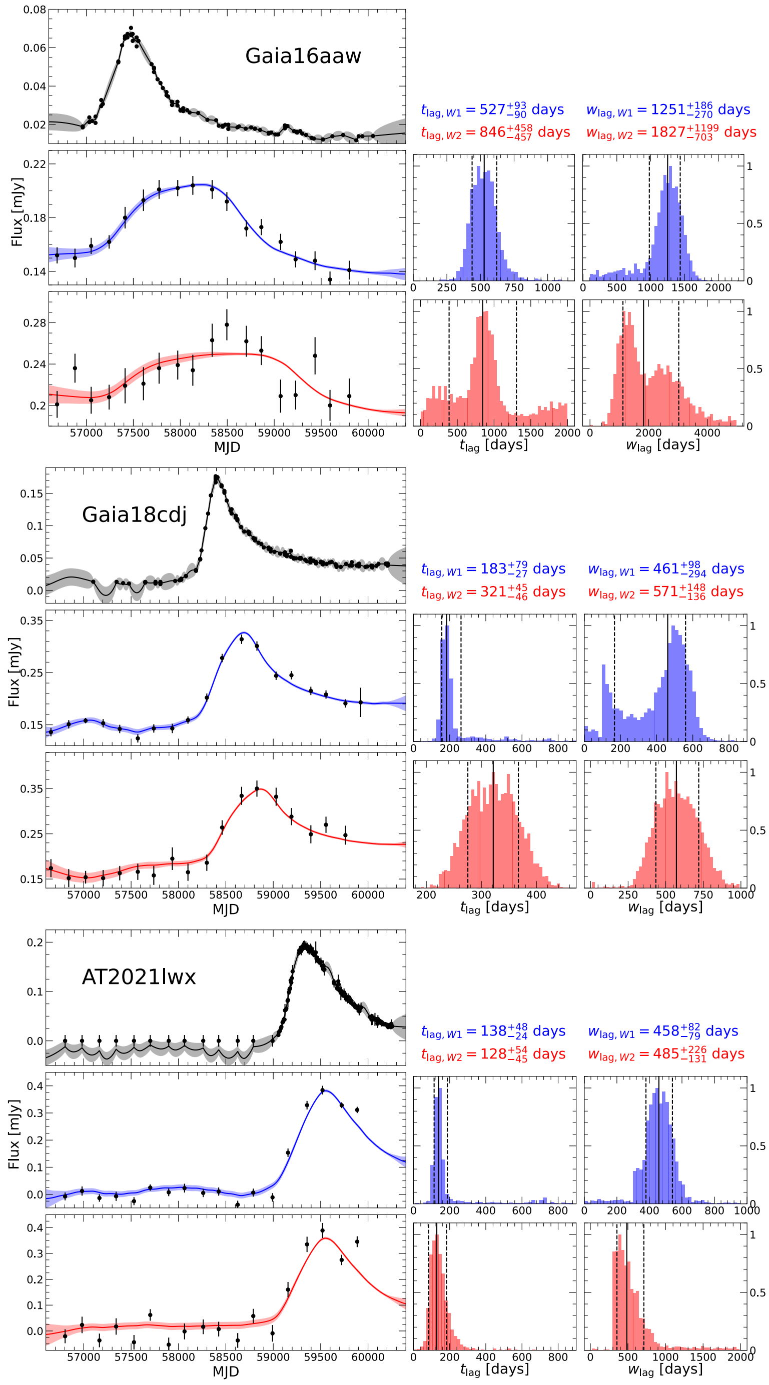

Figure S5: **JAVELIN (*199, 200*) fits to the observer-frame optical (black), NEOWISE $W1$ (blue), and NEOWISE $W2$ (red) light curves for Gaia16aaw (top panel), Gaia18cdj (middle panel), and AT2021lwx (bottom panel).** We use the Gaia G-band light curve for Gaia16aaw and Gaia18cdj and ZTF r-band light curve for AT2021lwx. The right panels within each figure give histograms for the lags (t_{lag}) and top hat smoothing length (W_{lag}).

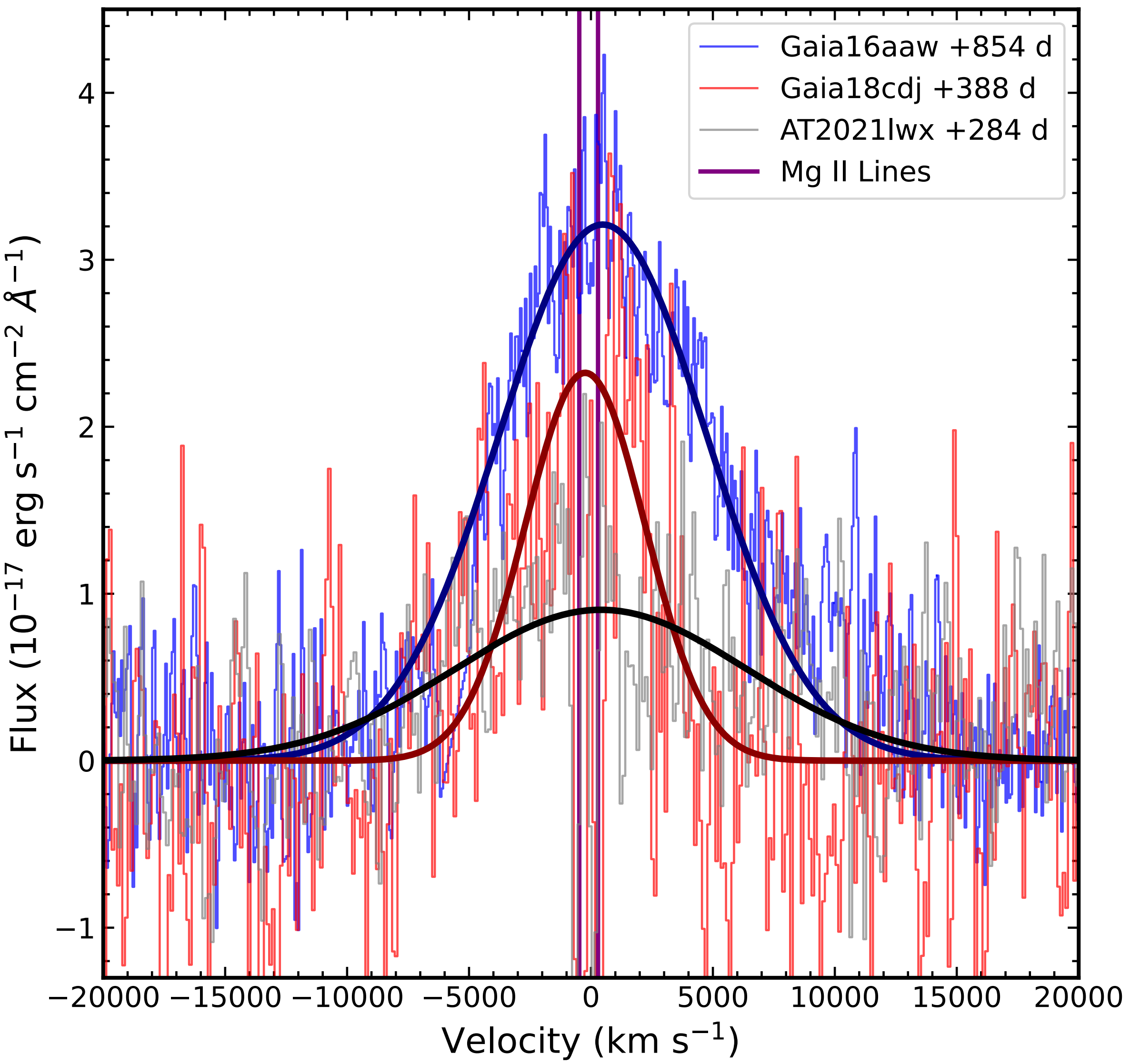

Figure S6: **The broad Mg II feature for Gaia16aaw (blue), Gaia18cdj (red), and AT2021lwx (gray).** Single-component Gaussian fits are shown as the solid line, with the color corresponding to the object.

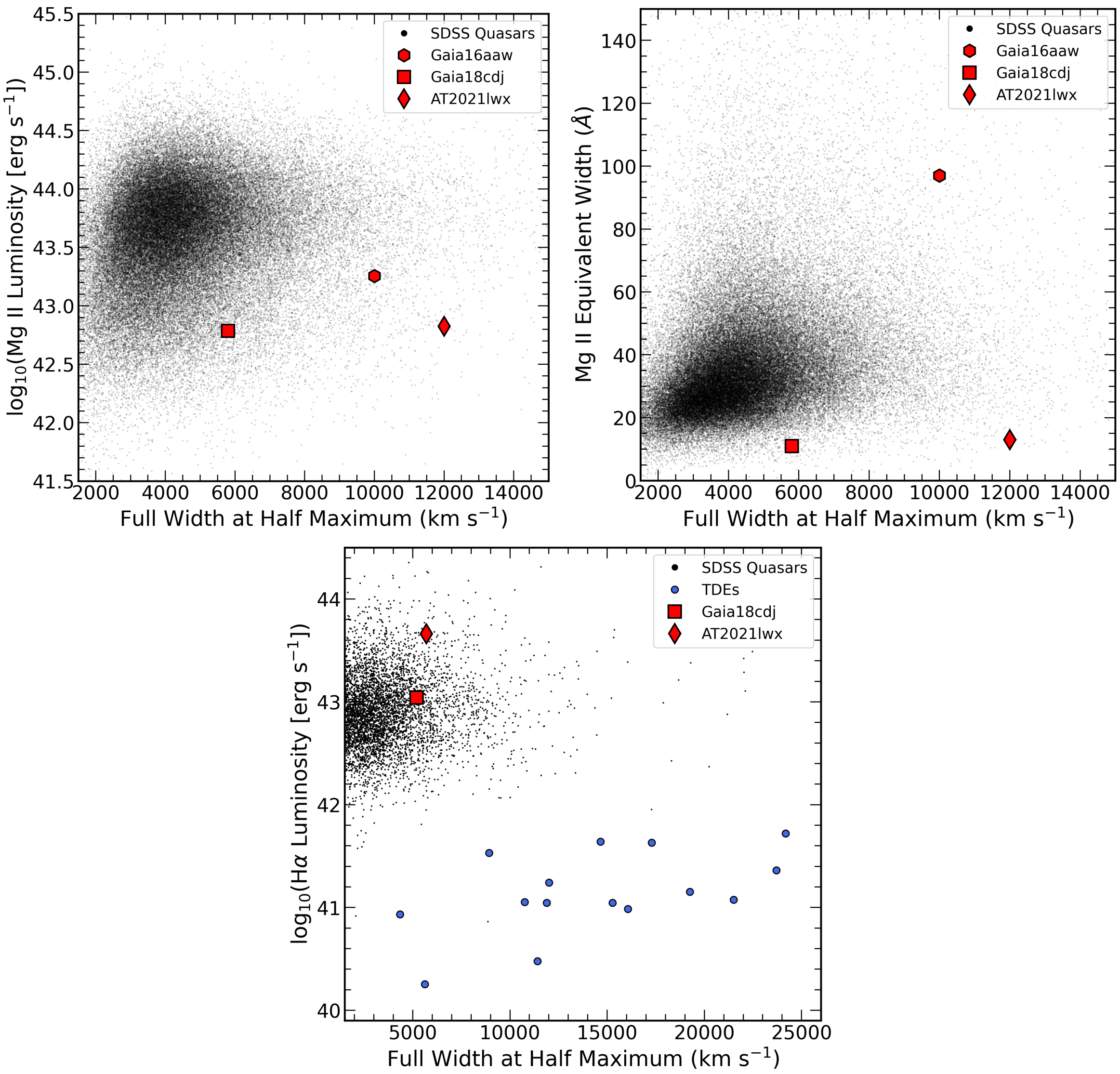

Figure S7: **ENT emission line parameters compared to AGNs and TDEs.** Mg II line luminosity as compared to FWHM (top left) and MG II equivalent width as compared to FWHM (top right) for the ENTs and a sample of SDSS quasars from (*123*). The bottom panel compares the Hα line luminosity to the FWHM again for the ENTs and SDSS quasars, along with TDEs from (*126*).

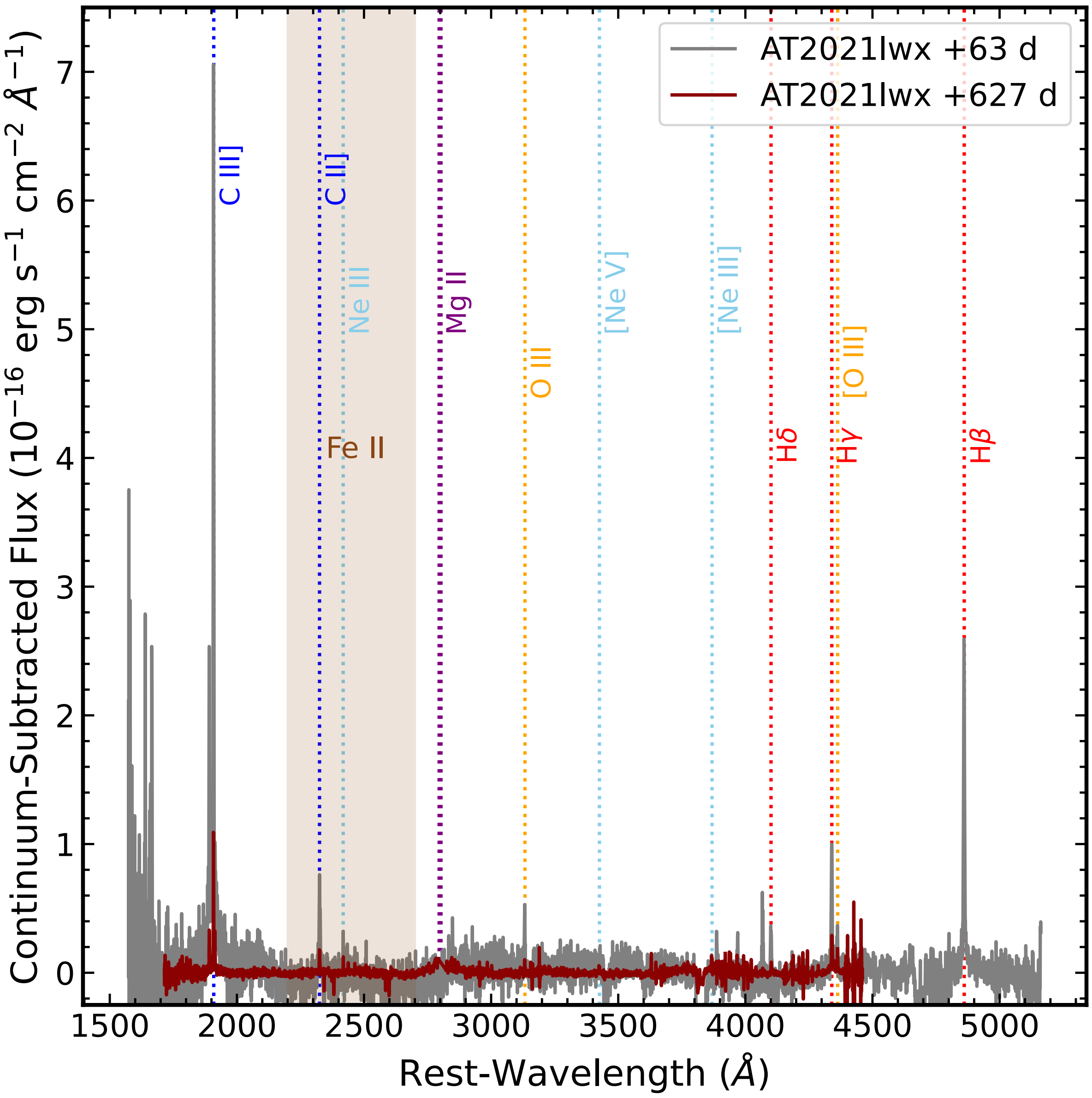

Figure S8: **Continuum-subtracted spectra taken of AT2021lwx at rest-frame phases of** $+63$ **d and** $+627$ **d.** Strong narrow lines of C, O, Ne, and H are seen both near peak and at late times. The emission line flux decrease is consistent with the decrease in the transient continuum.

Table S1: UV/optical Blackbody Fits

Object	Source	MJD	T_{16} (K)	T_{50} (K)	T_{84} (K)	L_{16} (erg/s)	L_{50} (erg/s)	L_{84} (erg/s)	R_{16} (cm)	R_{50} (cm)	R_{84} (cm)
Gaia16aaw	DES	57414.6[a]	4.33	4.34	4.36	45.26	45.28	45.30	15.51	15.53	15.54
Gaia16aaw	DES	57745.2[a]	4.25	4.26	4.27	44.92	44.93	44.94	15.50	15.51	15.53
Gaia16aaw	DES	58121.7[a]	4.17	4.18	4.19	44.18	44.19	44.20	15.29	15.31	15.33
Gaia18cdj	DES	58105.7[a]	3.92	3.93	3.94	44.39	44.40	44.41	15.89	15.91	15.93
Gaia18cdj	DES	58448.9[a]	4.27	4.28	4.29	45.64	45.66	45.67	15.82	15.84	15.85
Gaia18cdj	*Swift*	59164.3	4.33	4.36	4.39	45.06	45.10	45.14	15.33	15.42	15.49
AT2021lwx	*Swift*	59923.6	4.13	4.18	4.23	45.27	45.35	45.44	15.76	15.89	16.02
AT2021lwx	*Swift*	59966.5	4.20	4.25	4.30	45.17	45.26	45.34	15.56	15.70	15.83
AT2021lwx	*Swift*	60054.1	4.11	4.15	4.19	45.23	45.32	45.41	15.82	15.94	16.06
AT2021lwx	*Swift*	60067.1	4.10	4.21	4.33	44.83	45.08	45.50	15.32	15.69	16.13
AT2021lwx	*Swift*	60069.1	4.13	4.16	4.19	45.32	45.39	45.45	15.86	15.94	16.03
AT2021lwx	*Swift*	60100.5	4.12	4.15	4.18	45.20	45.27	45.33	15.82	15.90	15.98

UV/optical luminosity, effective radius, and temperature estimated from blackbody fits to the host-subtracted and extinction-corrected DES and/or *Swift* data. The values are given in common logarithms when scaled by the units in the second row of the header.

[a]The MJDs given for the DES epochs are the average of the MJDs for the individual bands used in that fit.

Table S2: X-ray Luminosities

Object	Source	MJD	log(L_x / (erg s^{-1}))	L_x Uncertainty
Gaia16aaw	ROSAT	48100.0	45.60	0.15
Gaia16aaw	ROSAT	50500.0	45.26	0.17
Gaia16aaw	*Swift*	59175.0	45.14	0.10
Gaia18cdj	ROSAT	48100.0	45.42	—
Gaia18cdj	*Swift*	59164.3	44.56	—
AT2021lwx	ROSAT	48200.0	46.02	—
AT2021lwx	*Swift*	59923.6	44.99	—
AT2021lwx	*Swift*	59966.5	44.93	0.24
AT2021lwx	*Swift*	60054.1	44.85	—
AT2021lwx	*Swift*	60067.1	44.59	0.20
AT2021lwx	*Swift*	60069.1	44.94	0.19
AT2021lwx	*Swift*	60100.5	44.68	0.23
AT2021lwx	XMM-Newton	60101.7	44.08	0.04
AT2021lwx	Chandra	60300.0	44.44	0.08

Rest-frame 0.3 - 10 keV X-ray luminosities for the ENTs in our sample. A value of "—" in the uncertainty column indicates a 3σ upper limit.

Table S3: Inputs for Rate Calculations

Survey	t_{survey} (yr)	Limiting Magnitude	f_{loss}
Gaia	9.5	20.7	1.0
ZTF	5.3	20.5	0.73

Time spans, limiting magnitudes, and loss factors corresponding to the survey coverage used to estimate the rates of the ENTs.

REFERENCES AND NOTES

1. F. Zou, Z. Yu, W. N. Brandt, H. Tak, G. Yang, Q. Ni, Mapping the growth of supermassive black holes as a function of galaxy stellar mass and redshift. *Astrophys. J.* **964**, 183 (2024).

2. C. L. MacLeod, Ž. Ivezić, B. Sesar, W. de Vries, C. S. Kochanek, B. C. Kelly, A. C. Becker, R. H. Lupton, P. B. Hall, G. T. Richards, S. F. Anderson, D. P. Schneider, A description of quasar variability measured using repeated SDSS and POSS imaging. *Astrophys. J.* **753**, 106 (2012).

3. B. J. Shappee, J. L. Prieto, D. Grupe, C. S. Kochanek, K. Z. Stanek, G. de Rosa, S. Mathur, Y. Zu, B. M. Peterson, R. W. Pogge, S. Komossa, M. Im, J. Jencson, T. W. S. Holoien, U. Basu, J. F. Beacom, D. M. Szczygieł, J. Brimacombe, S. Adams, A. Campillay, C. Choi, C. Contreras, M. Dietrich, M. Dubberley, M. Elphick, S. Foale, M. Giustini, C. Gonzalez, E. Hawkins, D. A. Howell, E. Y. Hsiao, M. Koss, K. M. Leighly, N. Morrell, D. Mudd, D. Mullins, J. M. Nugent, J. Parrent, M. M. Phillips, G. Pojmanski, W. Rosing, R. Ross, D. Sand, D. M. Terndrup, S. Valenti, Z. Walker, Y. Yoon, The man behind the curtain: X-rays drive the UV through NIR variability in the 2013 active galactic nucleus outburst in NGC 2617. *Astrophys. J.* **788**, 48 (2014).

4. K. D. Denney, G. de Rosa, K. Croxall, A. Gupta, M. C. Bentz, M. M. Fausnaugh, C. J. Grier, P. Martini, S. Mathur, B. M. Peterson, R. W. Pogge, B. J. Shappee, The typecasting of active galactic nuclei: Mrk 590 no longer fits the role. *Astrophys. J.* **796**, 134 (2014).

5. M. J. Graham, S. G. Djorgovski, A. J. Drake, D. Stern, A. A. Mahabal, E. Glikman, S. Larson, E. Christensen, Understanding extreme quasar optical variability with CRTS – I. Major AGN flares. *Mon. Not. R. Astron. Soc.* **470**, 4112–4132 (2017).

6. T. W.-S. Holoien, C. S. Kochanek, J. L. Prieto, K. Z. Stanek, S. Dong, B. J. Shappee, D. Grupe, J. S. Brown, U. Basu, J. F. Beacom, D. Bersier, J. Brimacombe, A. B. Danilet, E. Falco, Z. Guo, J. Jose, G. J. Herczeg, F. Long, G. Pojmanski, G. V. Simonian, D. M. Szczygieł, T. A. Thompson, J. R. Thorstensen, R. M. Wagner, P. R. Woźniak, Six months of multiwavelength follow-up of the tidal disruption candidate ASASSN-14li and implied TDE rates from ASAS-SN. *Mon. Not. R. Astron. Soc.* **455**, 2918–2935 (2016).

7. S. Gezari, Tidal disruption events. *Annu. Rev. Astron. Astrophys.* **59**, 21–58 (2021).

8. Ł. Wyrzykowski, M. Zieliński, Z. Kostrzewa-Rutkowska, A. Hamanowicz, P. G. Jonker, I. Arcavi, J. Guillochon, P. J. Brown, S. Kozłowski, A. Udalski, M. K. Szymański, I. Soszyński, R. Poleski, P. Pietrukowicz, J. Skowron, P. Mróz, K. Ulaczyk, M. Pawlak, K. A. Rybicki, J. Greiner, T. Krühler, J. Bolmer, S. J. Smartt, K. Maguire, K. Smith, OGLE16aaa – A signature of a hungry supermassive black hole. *Mon. Not. R. Astron. Soc.* **465**, L114–L118 (2017).
9. B. Trakhtenbrot, I. Arcavi, C. Ricci, S. Tacchella, D. Stern, H. Netzer, P. G. Jonker, A. Horesh, J. E. Mejía-Restrepo, G. Hosseinzadeh, V. Hallefors, D. A. Howell, C. McCully, M. Baloković, M. Heida, N. Kamraj, G. B. Lansbury, Ł. Wyrzykowski, M. Gromadzki, A. Hamanowicz, S. B. Cenko, D. J. Sand, E. Y. Hsiao, M. M. Phillips, T. R. Diamond, E. Kara, K. C. Gendreau, Z. Arzoumanian, R. Remillard, A new class of flares from accreting supermassive black holes. *Nat. Astron.* **3**, 242–250 (2019).

10. J. M. M. Neustadt, T. W. S. Holoien, C. S. Kochanek, K. Auchettl, J. S. Brown, B. J. Shappee, R. W. Pogge, S. Dong, K. Z. Stanek, M. A. Tucker, S. Bose, P. Chen, C. Ricci, P. J. Vallely, J. L. Prieto, T. A. Thompson, D. A. Coulter, M. R. Drout, R. J. Foley, C. D. Kilpatrick, A. L. Piro, C. Rojas-Bravo, D. A. H. Buckley, M. Gromadzki, G. Dimitriadis, M. R. Siebert, A. do, M. E. Huber, A. V. Payne, To TDE or not to TDE: The luminous transient ASASSN-18jd with TDE-like and AGN-like qualities. *Mon. Not. R. Astron. Soc.* **494**, 2538–2560 (2020).

11. S. Frederick, S. Gezari, M. J. Graham, J. Sollerman, S. van Velzen, D. A. Perley, D. Stern, C. Ward, E. Hammerstein, T. Hung, L. Yan, I. Andreoni, E. C. Bellm, D. A. Duev, M. Kowalski, A. A. Mahabal, F. J. Masci, M. Medford, B. Rusholme, R. Smith, R. Walters, A family tree of optical transients from narrow-line Seyfert 1 galaxies. *Astrophys. J.* **920**, 56 (2021).

12. J. T. Hinkle, T. W.-S. Holoien, B. J. Shappee, J. M. M. Neustadt, K. Auchettl, P. J. Vallely, M. Shahbandeh, M. Kluge, C. S. Kochanek, K. Z. Stanek, M. E. Huber, R. S. Post, D. Bersier, C. Ashall, M. A. Tucker, J. P. Williams, T. de Jaeger, A. Do, M. Fausnaugh, D. Gruen, U. Hopp, J. Myles, C. Obermeier, A. V. Payne, T. A. Thompson, The curious case of ASASSN-20hx: A slowly evolving, UV- and x-ray-luminous, ambiguous nuclear transient. *Astrophys. J.* **930**, 12 (2022).

13. J. T. Hinkle, T. W.-S. Holoien, B. J. Shappee, K. Auchettl, C. S. Kochanek, K. Z. Stanek, A. V. Payne, T. A. Thompson, Examining a peak-luminosity/decline-rate relationship for tidal disruption events. *Astrophys. J.* **894**, L10 (2020).

14. S. van Velzen, T. W.-S. Holoien, F. Onori, T. Hung, I. Arcavi, Optical-ultraviolet tidal disruption events. *Space Sci. Rev.* **216**, 124 (2020).

15. S. van Velzen, S. Gezari, E. Hammerstein, N. Roth, S. Frederick, C. Ward, T. Hung, S. B. Cenko, R. Stein, D. A. Perley, K. Taggart, R. J. Foley, J. Sollerman, N. Blagorodnova, I. Andreoni, E. C. Bellm, V. Brinnel, K. de, R. Dekany, M. Feeney, C. Fremling, M. Giomi, V. Z. Golkhou, M. J. Graham, A. Y. Q. Ho, M. M. Kasliwal, C. D. Kilpatrick, S. R. Kulkarni, T. Kupfer, R. R. Laher, A. Mahabal, F. J. Masci, A. A. Miller, J. Nordin, R. Riddle, B. Rusholme, J. Santen, Y. Sharma, D. L. Shupe, M. T. Soumagnac, Seventeen tidal disruption events from the first half of ZTF survey observations: Entering a new era of population studies. *Astrophys. J.* **908**, 4 (2021).

16. K. Auchettl, J. Guillochon, E. Ramirez-Ruiz, New physical insights about tidal disruption events from a comprehensive observational inventory at x-ray wavelengths. *Astrophys. J.* **838**, 149 (2017).

17. M. J. Rees, Tidal disruption of stars by black holes of 10^6-10^8 solar masses in nearby galaxies. *Nature* **333**, 523–528 (1988).

18. C. R. Evans, C. S. Kochanek, The tidal disruption of a star by a massive black hole. *Astrophys. J.* **346**, L13–L16 (1989).

19. B. Mockler, A. A. Twum, K. Auchettl, S. Dodd, K. D. French, J. A. P. Law-Smith, E. Ramirez-Ruiz, Evidence for the preferential disruption of moderately massive stars by supermassive black holes. *Astrophys. J.* **924**, 70 (2022).

20. T. Ryu, J. Krolik, T. Piran, Measuring stellar and black hole masses of tidal disruption events. *Astrophys. J.* **904**, 73 (2020).

21. C. S. Kochanek, Abundance anomalies in tidal disruption events. *Mon. Not. R. Astron. Soc.* **458**, 127–134 (2016).

22. S. Gomez, V. A. Villar, E. Berger, S. Gezari, S. van Velzen, M. Nicholl, P. K. Blanchard, K. D. Alexander, Identifying tidal disruption events with an expansion of the FLEET machine-learning algorithm. *Astrophys. J.* **949**, 113 (2023).

23. R. Stein, A. Mahabal, S. Reusch, M. Graham, M. M. Kasliwal, M. Kowalski, S. Gezari, E. Hammerstein, S. J. Nakoneczny, M. Nicholl, J. Sollerman, S. van Velzen, Y. Yao, R. R. Laher, B. Rusholme, tdescore: An accurate photometric classifier for tidal disruption events. *Astrophys. J.* **965**, L14 (2024).

24. F. Onori, G. Cannizzaro, P. G. Jonker, M. Kim, M. Nicholl, S. Mattila, T. M. Reynolds, M. Fraser, T. Wevers, E. Brocato, J. P. Anderson, R. Carini, P. Charalampopoulos, P. Clark, M. Gromadzki, C. P. Gutiérrez, N. Ihanec, C. Inserra, A. Lawrence, G. Leloudas, P. Lundqvist, T. E. Müller-Bravo, S. Piranomonte, M. Pursiainen, K. A. Rybicki, A. Somero, D. R. Young, K. C. Chambers, H. Gao, T. J. L. de Boer, E. A. Magnier, The nuclear transient AT 2017gge: A tidal disruption event in a dusty and gas-rich environment and the awakening of a dormant SMBH. *Mon. Not. R. Astron. Soc.* **517**, 76–98 (2022).

25. W. B. Hoogendam, J. T. Hinkle, B. J. Shappee, K. Auchettl, C. S. Kochanek, K. Z. Stanek, W. P. Maksym, M. A. Tucker, M. E. Huber, N. Morrell, C. R. Burns, D. Hey, T. W. S. Holoien, J. L. Prieto, M. Stritzinger, A. do, A. Polin, C. Ashall, P. J. Brown, J. M. DerKacy, L. Ferrari, L. Galbany, E. Y. Hsiao, S. Kumar, J. Lu, C. P. Stevens, Discovery and follow-up of ASASSN-23bd (AT 2023clx): The lowest redshift and luminosity optically selected tidal disruption event. *Mon. Not. R. Astron. Soc.* **530**, 4501–4518 (2024).

26. J. L. Tonry, L. Denneau, A. N. Heinze, B. Stalder, K. W. Smith, S. J. Smartt, C. W. Stubbs, H. J. Weiland, A. Rest, ATLAS: A high-cadence all-sky survey system. *Publ. Astron. Soc. Pac.* **130**, 064505 (2018).

27. S. T. Hodgkin, L. Wyrzykowski, N. Blagorodnova, S. Koposov, Transient astronomy with the Gaia satellite. *Philos. Trans. R. Soc. Lond. A* **371**, 20120239 (2013).

28. S. T. Hodgkin, D. L. Harrison, E. Breedt, T. Wevers, G. Rixon, A. Delgado, A. Yoldas, Z. Kostrzewa-Rutkowska, Ł.Wyrzykowski, M. van Leeuwen, N. Blagorodnova, H. Campbell, D. Eappachen, M. Fraser, N. Ihanec, S. E. Koposov, K. Kruszyńska, G. Marton, K. A. Rybicki, D. R. Young, Gaia Early Data Release 3: Gaia photometric science alerts. *Astron. Astrophys*. **652**, A76 (2021).

29. B. M. Subrayan, D. Milisavljevic, R. Chornock, R. Margutti, K. D. Alexander, V. Ramakrishnan, P. C. Duffell, D. A. Dickinson, K. S. Lee, D. Giannios, G. Lentner, M. Linvill, B. Garretson, M. J. Graham, D. Stern, D. Brethauer, T. Duong, W. Jacobson-Galán, N. LeBaron, D. Matthews, H. Sears, P. Venkatraman, Scary Barbie: An extremely energetic, long-duration tidal disruption event candidate without a detected host galaxy at z = 0.995. *Astrophys. J.* **948**, L19 (2023).

30. P. Wiseman, Y. Wang, S. Hönig, N. Castro-Segura, P. Clark, C. Frohmaier, M. D. Fulton, G. Leloudas, M. Middleton, T. E. Müller-Bravo, A. Mummery, M. Pursiainen, S. J. Smartt, K. Smith, M. Sullivan, J. P. Anderson, J. A. Acosta Pulido, P. Charalampopoulos, M. Banerji, M. Dennefeld, L. Galbany, M. Gromadzki, C. P. Gutiérrez, N. Ihanec, E. Kankare, A. Lawrence, B. Mockler, T. Moore, M. Nicholl, F. Onori, T. Petrushevska, F. Ragosta, S. Rest, M. Smith, T. Wevers, R. Carini, T. W. Chen, K. Chambers, H. Gao, M. Huber, C. Inserra, E. Magnier, L. Makrygianni, M. Toy, F. Vincentelli, D. R. Young, Multiwavelength observations of the extraordinary accretion event AT2021lwx. *Mon. Not. R. Astron. Soc.* **522**, 3992–4002 (2023).

31. D. Burgarella, V. Buat, J. Iglesias-Páramo, Star formation and dust attenuation properties in galaxies from a statistical ultraviolet-to-far-infrared analysis. *Mon. Not. R. Astron. Soc.* **360**, 1413–1425 (2005).

32. N. J. McConnell, C.-P. Ma, Revisiting the scaling relations of black hole masses and host galaxy properties. *Astrophys. J.* **764**, 184 (2013).

33. P. Madau, M. Dickinson, Cosmic star-formation dent. *Annu. Rev. Astron. Astrophys.* **52**, 415–486 (2014).

34. T. W.-S. Holoien, J. M. M. Neustadt, P. J. Vallely, K. Auchettl, J. T. Hinkle, C. Romero-Cañizales, B. J. Shappee, C. S. Kochanek, K. Z. Stanek, P. Chen, S. Dong, J. L. Prieto, T. A.

Thompson, T. G. Brink, A. V. Filippenko, W. K. Zheng, D. Bersier, S. Bose, A. J. Burgasser, S. Channa, T. de Jaeger, J. Hestenes, M. Im, B. Jeffers, H. D. Jun, G. Lansbury, R. S. Post, T. W. Ross, D. Stern, K. Tang, M. A. Tucker, S. Valenti, S. Yunus, K. D. Zhang, Investigating the nature of the luminous ambiguous nuclear transient ASASSN-17jz. *Astrophys. J.* **933**, 196 (2022).

35. E. Kankare, R. Kotak, S. Mattila, P. Lundqvist, M. J. Ward, M. Fraser, A. Lawrence, S. J. Smartt, W. P. S. Meikle, A. Bruce, J. Harmanen, S. J. Hutton, C. Inserra, T. Kangas, A. Pastorello, T. Reynolds, C. Romero-Cañizales, K. W. Smith, S. Valenti, K. C. Chambers, K. W. Hodapp, M. E. Huber, N. Kaiser, R. P. Kudritzki, E. A. Magnier, J. L. Tonry, R. J. Wainscoat, C. Waters, A population of highly energetic transient events in the centres of active galaxies. *Nat. Astron.* **1**, 865–871 (2017).

36. P. K. Blanchard, M. Nicholl, E. Berger, J. Guillochon, R. Margutti, R. Chornock, K. D. Alexander, J. Leja, M. R. Drout, PS16dtm: A tidal disruption event in a narrow-line Seyfert 1 galaxy. *Astrophys. J.* **843**, 106 (2017).

37. P. J. Pessi, R. Lunnan, J. Sollerman, S. Schulze, A. Gkini, A. Gangopadhyay, L. Yan, A. Gal-Yam, D. A. Perley, T.-W. Chen, K. R. Hinds, S. J. Brennan, Y. Hu, A. Singh, I. Andreoni, D. O Cook, C. Fremling, A. Y. Q. Ho, Y. Sharma, S. van Velzen, T. Kangas, A. Wold, E. C. Bellm, J. S. Bloom, M. J. Graham, M. M. Kasliwal, S. R. Kulkarni, R. Riddle, B. Rusholme, Sample of hydrogen-rich superluminous supernovae from the Zwicky Transient Facility arXiv:2408.15086 (2024).

38. S. Dong, B. J. Shappee, J. L. Prieto, S. W. Jha, K. Z. Stanek, T. W. S. Holoien, C. S. Kochanek, T. A. Thompson, N. Morrell, I. B. Thompson, U. Basu, J. F. Beacom, D. Bersier, J. Brimacombe, J. S. Brown, F. Bufano, P. Chen, E. Conseil, A. B. Danilet, E. Falco, D. Grupe, S. Kiyota, G. Masi, B. Nicholls, F. Olivares E., G. Pignata, G. Pojmanski, G. V. Simonian, D. M. Szczygiel, P. R. Woźniak, ASASSN-15lh: A highly super-luminous supernova. *Science* **351**, 257–260 (2016).

39. G. Leloudas, M. Fraser, N. C. Stone, S. van Velzen, P. G. Jonker, I. Arcavi, C. Fremling, J. R. Maund, S. J. Smartt, T. Krìhler, J. C. A. Miller-Jones, P. M. Vreeswijk, A. Gal-Yam, P. A. Mazzali, A. de Cia, D. A. Howell, C. Inserra, F. Patat, A. de Ugarte Postigo, O.

Yaron, C. Ashall, I. Bar, H. Campbell, T. W. Chen, M. Childress, N. Elias-Rosa, J. Harmanen, G. Hosseinzadeh, J. Johansson, T. Kangas, E. Kankare, S. Kim, H. Kuncarayakti, J. Lyman, M. R. Magee, K. Maguire, D. Malesani, S. Mattila, C. V. McCully, M. Nicholl, S. Prentice, C. Romero-Cañizales, S. Schulze, K. W. Smith, J. Sollerman, M. Sullivan, B. E. Tucker, S. Valenti, J. C. Wheeler, D. R. Young, The superluminous transient ASASSN-15lh as a tidal disruption event from a Kerr black hole. *Nat. Astron.* **1**, 0002 (2017).

40. S. L. West, R. Lunnan, C. M. B. Omand, T. Kangas, S. Schulze, N. L. Strotjohann, S. Yang, C. Fransson, J. Sollerman, D. Perley, L. Yan, T.-W. Chen, Z. H. Chen, K. Taggart, C. Fremling, J. S. Bloom, A. Drake, M. J. Graham, M. M. Kasliwal, R. Laher, M. S. Medford, J. D. Neill, R. Riddle, D. Shupe, SN 2020qlb: A hydrogen-poor superluminous supernova with well-characterized light curve undulations. *Astron. Astrophys.* **670**, A7 (2023).

41. Z. H. Chen, L. Yan, T. Kangas, R. Lunnan, S. Schulze, J. Sollerman, D. A. Perley, T.-W. Chen, K. Taggart, K. R. Hinds, A. Gal-Yam, X. F. Wang, I. Andreoni, E. Bellm, J. S. Bloom, K. Burdge, A. Burgos, D. Cook, A. Dahiwale, K. De, R. Dekany, A. Dugas, S. Frederik, C. Fremling, M. Graham, M. Hankins, A. Ho, J. Jencson, V. Karambelkar, M. Kasliwal, S. Kulkarni, R. Laher, B. Rusholme, Y. Sharma, F. Taddia, L. Tartaglia, B. P. Thomas, A. Tzanidakis, J. Van Roestel, R. Walter, Y. Yang, Y. H. Yao, O. Yaron, The hydrogen-poor superluminous supernovae from the Zwicky Transient Facility Phase I Survey. I. Light curves and measurements. *Astrophys. J.* **943**, 41 (2023).

42. X.-C. Ma, T.-G. Wang, The covering factor of warm dust in quasars: A view from the Wide-field Infrared Survey Explorer All-Sky Data Release. *Mon. Not. R. Astron. Soc.* **430**, 3445–3452 (2013).

43. S. van Velzen, A. J. Mendez, J. H. Krolik, V. Gorjian, Discovery of transient infrared emission from dust heated by stellar tidal disruption flares. *Astrophys. J.* **829**, 19 (2016).

44. M. Masterson, K. De, C. Panagiotou, E. Kara, I. Arcavi, A.-C. Eilers, D. Frostig, S. Gezari, I. Grotova, Z. Liu, A. Malyali, A. M. Meisner, A. Merloni, M. Newsome, A. Rau, R. A. Simcoe, S. van Velzen, A new population of mid-infrared-selected tidal disruption events:

Implications for tidal disruption event rates and host galaxy properties. *Astrophys. J.* **961**, 211 (2024).

45. C. Ricci, B. Trakhtenbrot, M. J. Koss, Y. Ueda, I. Del Vecchio, E. Treister, K. Schawinski, S. Paltani, K. Oh, I. Lamperti, S. Berney, P. Gandhi, K. Ichikawa, F. E. Bauer, L. C. Ho, D. Asmus, V. Beckmann, S. Soldi, M. Baloković, N. Gehrels, C. B. Markwardt, BAT AGN Spectroscopic Survey. V. X-ray properties of the Swift/BAT 70-month AGN catalog. *Astrophys. J.* **233**, 17 (2017).

46. S. Prajs, M. Sullivan, M. Smith, A. Levan, N. V. Karpenka, T. D. P. Edwards, C. R. Walker, W. M. Wolf, C. Balland, R. Carlberg, D. A. Howell, C. Lidman, R. Pain, C. Pritchet, V. Ruhlmann-Kleider, The volumetric rate of superluminous supernovae at z ~ 1. *Mon. Not. R Astron. Soc.* **464**, 3568–3579 (2017).

47. C. S. Kochanek, Tidal disruption event demographics. *Mon. Not. R. Astron. Soc.* **461**, 371–384 (2016).

48. P. L. Kelly, S. A. Rodney, T. Treu, R. J. Foley, G. Brammer, K. B. Schmidt, A. Zitrin, A. Sonnenfeld, L. G. Strolger, O. Graur, A. V. Filippenko, S. W. Jha, A. G. Riess, M. Bradac, B. J. Weiner, D. Scolnic, M. A. Malkan, A. von der Linden, M. Trenti, J. Hjorth, R. Gavazzi, A. Fontana, J. C. Merten, C. McCully, T. Jones, M. Postman, A. Dressler, B. Patel, S. B. Cenko, M. L. Graham, B. E. Tucker, Multiple images of a highly magnified supernova formed by an early-type cluster galaxy lens. *Science* **347**, 1123–1126 (2015).

49. E. L. Turner, The effect of undetected gravitational lenses on statistical measures of quasar evolution. *Astrophys. J.* **242**, L135–L139 (1980).

50. A. Gal-Yam, Luminous supernovae. *Science* **337**, 927–932 (2012).

51. R. C. Tolman, Static solutions of Einstein's field equations for spheres of fluid. *Phys. Rev.* **55**, 364–373 (1939).

52. T. J. Moriya, M. Tanaka, T. Morokuma, K. Ohsuga, Superluminous transients at AGN centers from interaction between black hole disk winds and broad-line region clouds. *Astrophys. J. Lett.* **843**, L19 (2017).

53. N. Stone, R. Sari, A. Loeb, Consequences of strong compression in tidal disruption events. *Mon. Not. R. Astron. Soc.* **435**, 1809–1824 (2013).

54. J. R. Lu, T. Do, A. M. Ghez, M. R. Morris, S. Yelda, K. Matthews, Stellar populations in the central 0.5 pc of the galaxy. II. The initial mass function. *Astrophys. J.* **764**, 155 (2013).

55. B. Mockler, J. Guillochon, E. Ramirez-Ruiz, Weighing black holes using tidal disruption events. *Astrophys. J.* **872**, 151 (2019).

56. D. J. Price, D. Liptai, I. Mandel, J. Shepherd, G. Lodato, Y. Levin, Eddington envelopes: The fate of stars on parabolic orbits tidally disrupted by supermassive black holes. *Astrophys. J. Lett.* **971**, L46 (2024).

57. Y. Wang, D. N. C. Lin, B. Zhang, Z. Zhu, Changing-look active galactic nuclei behavior induced by disk-captured tidal disruption events. *Astrophys. J. Lett.* **962**, L7 (2024).

58. K. Kaur, N. C. Stone, Elevated rates of tidal disruption events in active galactic nuclei. *Astrophys. J.* **979**, 172 (2025).

59. Ž. Ivezić, S. M. Kahn, J. Anthony Tyson, B. Abel, E. Acosta, R. Allsman, D. Alonso, Y. A. Sayyad, S. F. Anderson, J. Andrew, J. R. P. Angel, G. Z. Angeli, R. Ansari, P. Antilogus, Araujo, R. Armstrong, K. T. Arndt, P. Astier, É. Aubourg, N. Auza, T. S. Axelrod, D. J. Bard, J. D. Barr, A. Barrau, J. G. Bartlett, A. E. Bauer, B. J. Bauman, S. Baumont, A. C. Becker, J. Becla, C. Beldica, S. Bellavia, F. B. Bianco, R. Biswas, G. Blanc, J. Blazek, R. D. Blandford, J. S. Bloom, J. Bogart, T. W. Bond, A. W. Borgland, K. Borne, J. F. Bosch, D. Boutigny, C. A. Brackett, A. Bradshaw, W. N. Brandt, M. E. Brown, J. S. Bullock, P. Burchat, D. L. Burke, G. Cagnoli, D. Calabrese, S. Callahan, A. L. Callen, S. Chandrasekharan, G. Charles-Emerson, S. Chesley, E. C. Cheu, H.-F. Chiang, J. Chiang, C. Chirino, D. Chow, D. R. Ciardi, C. F. Claver, J. Cohen-Tanugi, J. J. Cockrum, R. Coles, A. J. Connolly, K. H. Cook, A. Cooray, K. R. Covey, C. Cribbs, W. Cui, R. Cutri, P. N. Daly, S. F. Daniel, F. Daruich, G. Daubard, G.

Daues, W. Dawson, F. Delgado, A. Dellapenna, R. de Peyster, Miguel de Val-Borro, S. W. Digel, P. Doherty, R. Dubois, G. P. Dubois-Felsmann, J. Durech, F. Economou, M. Eracleous, H. Ferguson, E. Figueroa, M. Fisher-Levine, W. Focke, M. D. Foss, J. Frank, M. D. Freemon, E. Gangler, LSST Collaboration, LSST: From science drivers to reference design and anticipated data products. arXiv:0805.2366 (2008).

60. P. F. Hopkins, G. T. Richards, L. Hernquist, An observational determination of the bolometric quasar luminosity function. *Astrophys. J.* **654**, 731–753 (2007).

61. D. Spergel, N. Gehrels, C. Baltay, D. Bennett, J. Breckinridge, M. Donahue, A. Dressler, B. S. Gaudi, T. Greene, O. Guyon, C. Hirata, J. Kalirai, N. J. Kasdin, B. Macintosh, W. Moos, S. Perlmutter, M. Postman, B. Rauscher, J. Rhodes, Y. Wang, D. Weinberg, D. Benford, M. Hudson, W. -S. Jeong, Y. Mellier, W. Traub, T. Yamada, P. Capak, J. Colbert, D. Masters, M. Penny, D. Savransky, D. Stern, N. Zimmerman, R. Barry, L. Bartusek, K. Carpenter, E. Cheng, D. Content, F. Dekens, R. Demers, K. Grady, C. Jackson, G. Kuan, J. Kruk, M. Melton, B. Nemati, B. Parvin, I. Poberezhskiy, C. Peddie, J. Ruffa, J. K. Wallace, A. Whipple, E. Wollack, F. Zhao, Wide-Field InfrarRed Survey Telescope-astrophysics focused telescope assets WFIRST-AFTA 2015 report. arXiv:1503.03757 (2015).

62. A. J. Drake, S. G. Djorgovski, A. Mahabal, E. Beshore, S. Larson, M. J. Graham, R. Williams, E. Christensen, M. Catelan, A. Boattini, A. Gibbs, R. Hill, R. Kowalski, First results from the Catalina Real-Time Transient Survey. *Astrophys. J.* **696**, 870–884 (2009).

63. E. L. Wright, P. R. M. Eisenhardt, A. K. Mainzer, M. E. Ressler, R. M. Cutri, T. Jarrett, J. D. Kirkpatrick, D. Padgett, R. S. Mc Millan, M. Skrutskie, S. A. Stanford, M. Cohen, R. G. Walker, J. C. Mather, D. Leisawitz, T. N. Gautier III, I. M. Lean, D. Benford, C. J. Lonsdale, A. Blain, B. Mendez, W. R. Irace, V. Duval, F. Liu, D. Royer, I. Heinrichsen, J. Howard, M. Shannon, M. Kendall, A. L. Walsh, M. Larsen, J. G. Cardon, S. Schick, M. Schwalm, M. Abid, B. Fabinsky, L. Naes, C.-W. Tsai, The Wide-field Infrared Survey Explorer (WISE): Mission description and initial on-orbit performance. *Astron. J.* **140**, 1868 (2010).

64. The Dark Energy Survey Collaboration, The Dark Energy Survey. arXiv:astro-ph/0510346 (2005).

65. N. Gehrels, G. Chincarini, P. Giommi, K. O. Mason, J. A. Nousek, A. A. Wells, N. E. White, S. D. Barthelmy, D. N. Burrows, L. R. Cominsky, K. C. Hurley, F. E. Marshall, P. Meszaros, P. W. A. Roming, L. Angelini, L. M. Barbier, T. Belloni, S. Campana, P. A. Caraveo, M. M. Chester, O. Citterio, T. L. Cline, M. S. Cropper, J. R. Cummings, A. J. Dean, E. D. Feigelson, E. E. Fenimore, D. A. Frail, A. S. Fruchter, G. P. Garmire, K. Gendreau, G. Ghisellini, J. Greiner, J. E. Hill, S. D. Hunsberger, H. A. Krimm, S. R. Kulkarni, P. Kumar, F. Lebrun, N. M. Lloyd Ronning, C. B. Markwardt, B. J. Mattson, R. F. Mushotzky, J. P. Norris, J. Osborne, B. Paczynski, D. M. Palmer, H. S. Park, A. M. Parsons, J. Paul, M. J. Rees, C. S. Reynolds, J. E. Rhoads, T. P. Sasseen, B. E. Schaefer, A. T. Short, A. P. Smale, I. A. Smith, L. Stella, G. Tagliaferri, T. Takahashi, M. Tashiro, L. K. Townsley, J. Tueller, M. J. L. Turner, M. Vietri, W. Voges, M. J. Ward, R. Willingale, F. M. Zerbi, W. W. Zhang, The Swift Gamma-Ray Burst Mission. *Astrophys. J.* **611**, 1005–1020 (2004).

66. C. Alard, R. H. Lupton, A method for optimal image subtraction. *Astrophys. J.* **503**, 325 (1998).

67. C. Alard, Image subtraction using a space-varying kernel. *Astron. Astrophys.* **144**, 363–370 (2000).

68. E. Bertin, S. Arnouts, SExtractor: Software for source extraction. *Astron. Astrophys. Suppl. Ser.* **117**, 393–404 (1996).

69. R. J. Siverd, T. G. Beatty, J. Pepper, J. D. Eastman, K. Collins, A. Bieryla, D. W. Latham, L. A. Buchhave, E. L. N. Jensen, J. R. Crepp, R. Street, K. G. Stassun, B. S. Gaudi, P. Berlind, M. L. Calkins, D. L. De Poy, G. A. Esquerdo, B. J. Fulton, G. Fűrész, J. C. Geary, A. Gould, L. Hebb, J. F. Kielkopf, J. L. Marshall, R. Pogge, K. Z. Stanek, R. P. Stefanik, A. H. Szentgyorgyi, M. Trueblood, P. Trueblood, A. M. Stutz, J. L. van Saders, KELT-1b: A strongly irradiated, highly inflated, short period, 27 Jupiter-mass companion transiting a mid-F star. *Astrophys. J.* **761**, 123 (2012).

70. L. Bradley, B. Sipőcz, T. Robitaille, E. Tollerud, Z. Vinícius, C. Deil, K. Barbary, T. J. Wilson, I. Busko, A. Donath, H. M. Günther, M. Cara, P. L. Lim, S. Meßlinger, S. Conseil, Z. Burnett, A. Bostroem, M. Droettboom, E. M. Bray, L. A. Bratholm, W. Jamieson, A. Ginsburg, G. Barentsen, M. Craig, astropy/photutils: 1.10.0 (2023).

71. A. Dressler, B. Bigelow, T. Hare, B. Sutin, I. Thompson, G. Burley, H. Epps, A. Oemler Jr., A. Bagish, C. Birk, K. Clardy, S. Gunnels, D. Kelson, S. Shectman, D. Osip, IMACS: The Inamori-Magellan Areal Camera and Spectrograph on Magellan-Baade. *Publ. Astron. Soc. Pac.* **123**, 288–332 (2011).

72. J. C. Clemens, J. A. Crain, R. Anderson, "The Goodman spectrograph" in *Ground-based Instrumentation for Astronomy*, A. F. M. Moorwood, M. Iye, Eds., vol. 5492 (SPIE, 2004), pp. 331–340.

73. D. Tody, "The IRAF data reduction and analysis system" in *Instrumentation in Astronomy VI*, D. L. Crawford, Ed., vol. 627 of *Society of Photo-Optical Instrumentation Engineers (SPIE) Conference Series* (SPIE, 1986), p. 733.

74. D. Tody, "IRAF in the Nineties" in *Astronomical Data Analysis Software and Systems II*, R. J. Hanisch, R. J. V. Brissenden, J. Barnes, Eds., vol. 52 of *Astronomical Society of the Pacific Conference Series* (Astronomical Society of the Pacific, 1993), p. 173.

75. J. Prochaska, J. F. Hennawi, K. B. Westfall, R. J. Cooke, F. Wang, T. Hsyu, F. B. Davies, E. P. Farina, PypeIt: The Python spectroscopic data reduction pipeline. *J. Open Source Softw.* **5**, 2308 (2020).

76. P. Morrissey, M. Matuszewski, D. C. Martin, J. D. Neill, H. Epps, J. Fucik, B. Weber, B. Darvish, S. Adkins, S. Allen, R. Bartos, J. Belicki, J. Cabak, S. Callahan, D. Cowley, M. Crabill, W. Deich, A. Delecroix, G. Doppman, D. Hilyard, E. James, S. Kaye, M. Kokorowski, S. Kwok, K. Lanclos, S. Milner, A. Moore, D. O'Sullivan, P. Parihar, S. Park, A. Phillips, L. Rizzi, C. Rockosi, H. Rodriguez, Y. Salaun, K. Seaman, D. Sheikh, J. Weiss, R. Zarzaca, The Keck Cosmic Web Imager integral field spectrograph. *Astrophys. J.* **864**, 93 (2018).

77. R. A. Simcoe, A. J. Burgasser, R. A. Bernstein, B. C. Bigelow, J. Fishner, W. J. Forrest, C. McMurtry, J. L. Pipher, P. L. Schechter, M. Smith, "FIRE: A near-infrared cross-dispersed echellette spectrometer for the Magellan telescopes" in *Ground-based and Airborne Instrumentation for Astronomy II*, I. S. McLean, M. M. Casali, Eds., vol. 7014 of *Society of*

Photo-Optical Instrumentation Engineers (SPIE) Conference Series (SPIE, 2008), p. 70140U.

78. M. Boquien, D. Burgarella, Y. Roehlly, V. Buat, L. Ciesla, D. Corre, A. K. Inoue, H. Salas, CIGALE: A Python Code Investigating GALaxy Emission. *Astron. Astrophys.* **622**, A103 (2019).

79. G. Yang, M. Boquien, W. N. Brandt, V. Buat, D. Burgarella, L. Ciesla, B. D. Lehmer, K. Małek, G. Mountrichas, C. Papovich, E. Pons, M. Stalevski, P. Theulé, S. Zhu, Fitting AGN/galaxy x-ray-to-radio SEDs with CIGALE and improvement of the code. *Astrophys. J.* **927**, 192 (2022).

80. R. G. McMahon, M. Banerji, E. Gonzalez, S. E. Koposov, V. J. Bejar, N. Lodieu, R. Rebolo, VHS collaboration, First scientific results from the VISTA Hemisphere Survey (VHS). *Messenger* **154**, 35–37 (2013).

81. G. Bruzual, S. Charlot, Stellar population synthesis at the resolution of 2003. *Mon. Not. R. Astron. Soc.* **344**, 1000–1028 (2003).

82. E. E. Salpeter, The luminosity function and stellar evolution. *Astrophys. J.* **121**, 161 (1955).

83. J. A. Cardelli, G. C. Clayton, J. S. Mathis, The relationship between infrared, optical, and ultraviolet extinction. *Astrophys. J.* **345**, 245–256 (1989).

84. M. Stalevski, J. Fritz, M. Baes, T. Nakos, L. Č. Popović, 3D radiative transfer modelling of the dusty tori around active galactic nuclei as a clumpy two-phase medium. *Mon. Not. R. Astron. Soc.* **420**, 2756–2772 (2012).

85. M. Stalevski, C. Ricci, Y. Ueda, P. Lira, J. Fritz, M. Baes, The dust covering factor in active galactic nuclei. *Mon. Not. R. Astron. Soc.* **458**, 2288–2302 (2016).

86. E. F. Bell, R. S. de Jong, Stellar mass-to-light ratios and the Tully-Fisher relation. *Astrophys. J.* **550**, 212–229 (2001).

87. J. T. Hinkle, T. W. S. Holoien, B. J. Shappee, K. Auchettl, A swift fix for nuclear outbursts. *Astrophys. J.* **910**, 83 (2021).

88. D. J. Eisenstein, D. H. Weinberg, E. Agol, H. Aihara, C. A. Prieto, S. F. Anderson, J. A. Arns, É. Aubourg, S. Bailey, E. Balbinot, R. Barkhouser, T. C. Beers, A. A. Berlind, S. J. Bickerton, D. Bizyaev, M. R. Blanton, J. J. Bochanski, A. S. Bolton, C. T. Bosman, J. Bovy, W. N. Brandt, B. Breslauer, H. J. Brewington, J. Brinkmann, P. J. Brown, J. R. Brownstein, D. Burger, N. G. Busca, H. Campbell, P. A. Cargile, W. C. Carithers, J. K. Carlberg, M. A. Carr, L. Chang, Y. Chen, C. Chiappini, J. Comparat, N. Connolly, M. Cortes, R. A. C. Croft, K. Cunha, L. N. da Costa, J. R. A. Davenport, K. Dawson, N. De Lee, G. F. Porto de Mello, F. de Simoni, J. Dean, S. Dhital, A. Ealet, G. L. Ebelke, E. M. Edmondson, J. M. Eiting, S. Escoffier, M. Esposito, M. L. Evans, X. Fan, B. F. Castellá, L. D. Ferreira, G. Fitzgerald, S. W. Fleming, A. Font-Ribera, E. B. Ford, P. M. Frinchaboy, A. E. G. Pérez, B. S. Gaudi, J. Ge, L. Ghezzi, B. A. Gillespie, G. Gilmore, L. Girardi, J. R. Gott, A. Gould, E. K. Grebel, J. E. Gunn, J.-C. Hamilton, P. Harding, D. W. Harris, S. L. Hawley, F. R. Hearty, J. F. Hennawi, J. I. González Hernández, S. Ho, D. W. Hogg, J. A. Holtzman, K. Honscheid, N. Inada, I. I. Ivans, L. Jiang, P. Jiang, J. A. Johnson, C. Jordan, W. P. Jordan, G. Kauffmann, E. Kazin, D. Kirkby, M. A. Klaene, G. R. Knapp, J.-P. Kneib, C. S. Kochanek, L. Koesterke, J. A. Kollmeier, R. G. Kron, H. Lampeitl, D. Lang, J. E. Lawler, J.-M. Le Goff, B. L. Lee, Y. S. Lee, J. M. Leisenring, Y.-T. Lin, J. Liu, D. C. Long, C. P. Loomis, S. Lucatello, B. Lundgren, R. H. Lupton, B. Ma, Z. Ma, N. M. Donald, C. Mack, S. Mahadevan, M. A. G. Maia, S. R. Majewski, M. Makler, E. Malanushenko, V. Malanushenko, R. Mandelbaum, C. Maraston, D. Margala, P. Maseman, K. L. Masters, C. K. Mc Bride, P. M. Donald, I. D. Mc Greer, R. G. Mc Mahon, O. M. Requejo, B. Ménard, J. Miralda-Escudé, H. L. Morrison, F. Mullally, D. Muna, H. Murayama, A. D. Myers, T. Naugle, A. F. Neto, D. C. Nguyen, R. C. Nichol, D. L. Nidever, R. W. O'Connell, R. L. C. Ogando, M. D. Olmstead, D. J. Oravetz, N. Padmanabhan, M. Paegert, N. Palanque-Delabrouille, K. Pan, P. Pandey, J. K. Parejko, I. Pâris, P. Pellegrini, J. Pepper, W. J. Percival, P. Petitjean, R. Pfaffenberger, J. Pforr, S. Phleps, C. Pichon, M. M. Pieri, F. Prada, A. M. Price-Whelan, M. J. Raddick, B. H. F. Ramos, I. N. Reid, C. Reyle, J. Rich, G. T. Richards, G. H. Rieke, M. J. Rieke, H.-W. Rix, A. C. Robin, H. J. Rocha-Pinto, C. M. Rockosi, N. A. Roe, E. Rollinde, A. J. Ross, N. P. Ross, B. Rossetto, A. G. Sánchez, B. Santiago, C. Sayres, R. Schiavon, D. J. Schlegel, K. J. Schlesinger, S. J. Schmidt, D. P. Schneider, K. Sellgren, A. Shelden, E. Sheldon, M. Shetrone, Y. Shu, J. D. Silverman, J. Simmerer, A. E. Simmons, T. Sivarani, M. F. Skrutskie, A. Slosar, S. Smee, V. V. Smith, S. A. Snedden, K. G. Stassun, O. Steele, M. Steinmetz, M. H. Stockett, T. Stollberg,

M. A. Strauss, A. S. Szalay, M. Tanaka, A. R. Thakar, D. Thomas, J. L. Tinker, B. M. Tofflemire, R. Tojeiro, C. A. Tremonti, M. V. Magaña, L. Verde, N. P. Vogt, D. A. Wake, X. Wan, J. Wang, B. A. Weaver, M. White, S. D. M. White, J. C. Wilson, J. P. Wisniewski, W. M. Wood-Vasey, B. Yanny, N. Yasuda, C. Yèche, D. G. York, E. Young, G. Zasowski, I. Zehavi, B. Zhao, SDSS-III: Massive spectroscopic surveys of the distant universe, the Milky Way, and extra-solar planetary systems. *Astron. J.* **142**, 72 (2011).

89. J. Brinchmann, S. Charlot, S. D. M. White, C. Tremonti, G. Kauffmann, T. Heckman, J. Brinkmann, The physical properties of star-forming galaxies in the low-redshift Universe. *Mon. Not. R. Astron. Soc.* **351**, 1151–1179 (2004).

90. J. R. Weaver, O. B. Kauffmann, O. Ilbert, H. J. McCracken, A. Moneti, S. Toft, G. Brammer, M. Shuntov, I. Davidzon, B. C. Hsieh, C. Laigle, A. Anastasiou, C. K. Jespersen, J. Vinther, P. Capak, C. M. Casey, C. J. R. M. Partland, B. Milvang-Jensen, B. Mobasher, D. B. Sanders, L. Zalesky, S. Arnouts, H. Aussel, J. S. Dunlop, A. Faisst, M. Franx, L. J. Furtak, J. P. U. Fynbo, K. M. L. Gould, T. R. Greve, S. Gwyn, J. S. Kartaltepe, D. Kashino, A. M. Koekemoer, V. Kokorev, O. Le Fèvre, S. Lilly, D. Masters, G. Magdis, V. Mehta, Y. Peng, D. A. Riechers, M. Salvato, M. Sawicki, C. Scarlata, N. Scoville, R. Shirley, J. D. Silverman, A. Sneppen, V. Smolčić, C. Steinhardt, D. Stern, M. Tanaka, Y. Taniguchi, H. I. Teplitz, M. Vaccari, W.-H. Wang, G. Zamorani, COSMOS2020: A panchromatic view of the universe to z~10 from two complementary catalogs. *Astrophys. J. Suppl. Ser.* **258**, 11 (2022).

91. P. Popesso, A. Concas, G. Cresci, S. Belli, G. Rodighiero, H. Inami, M. Dickinson, O. Ilbert, M. Pannella, D. Elbaz, The main sequence of star-forming galaxies across cosmic times. *Mon. Not. R. Astron. Soc.* **519**, 1526–1544 (2023).

92. K. Guo, X. Z. Zheng, H. Fu, The intrinsic scatter along the main sequence of star-forming galaxies at z □ 0.7. *Astrophys. J.* **778**, 23 (2013).

93. J. T. Mendel, L. Simard, M. Palmer, S. L. Ellison, D. R. Patton, A catalog of bulge, disk, and total stellar mass estimates for the Sloan Digital Sky Survey. *Astrophys. J. Suppl. Ser.* **210**, 3 (2014).

94. R. J. Assef, C. S. Kochanek, M. Brodwin, R. Cool, W. Forman, A. H. Gonzalez, R. C. Hickox, C. Jones, E. Le Floc'h, J. Moustakas, S. S. Murray, D. Stern, Low-resolution spectral templates for active galactic nuclei and galaxies from 0.03 to 30 μm. *Astrophys. J.* **713**, 970–985 (2010).

95. R. J. Assef, D. Stern, C. S. Kochanek, A. W. Blain, M. Brodwin, M. J. I. Brown, E. Donoso, P. R. M. Eisenhardt, B. T. Jannuzi, T. H. Jarrett, S. A. Stanford, C.-W. Tsai, J. Wu, L. Yan, Mid-infrared selection of active galactic nuclei with the Wide-field Infrared Survey Explorer. II. Properties of WISE-selected active galactic nuclei in the NDWFS Boötes Field. *Astrophys. J.* **772**, 26 (2013).

96. N. L. Zakamska, M. A. Strauss, J. H. Krolik, M. J. Collinge, P. B. Hall, L. Hao, T. M. Heckman, Z. Ivezić, G. T. Richards, D. J. Schlegel, D. P. Schneider, I. Strateva, D. E. V. Berk, S. F. Anderson, J. Brinkmann, Candidate type II quasars from the Sloan Digital Sky Survey. I. Selection and optical properties of a sample at 0.3<Z<0.83. *Astrophys. J.* **126**, 2125–2144 (2003).

97. L. Hao, M. A. Strauss, X. Fan, C. A. Tremonti, D. J. Schlegel, T. M. Heckman, G. Kauffmann, M. R. Blanton, J. E. Gunn, P. B. Hall, Ž. Ivezić, G. R. Knapp, J. H. Krolik, R. H. Lupton, G. T. Richards, D. P. Schneider, I. V. Strateva, N. L. Zakamska, J. Brinkmann, G. P. Szokoly, Active galactic nuclei in the Sloan Digital Sky Survey. II. Emission-line luminosity function. *Astron. J.* **129**, 1795–1808 (2005).

98. A. Bongiorno, M. Mignoli, G. Zamorani, F. Lamareille, G. Lanzuisi, T. Miyaji, M. Bolzonella, C. M. Carollo, T. Contini, J. P. Kneib, O. Le Fèvre, S. J. Lilly, V. Mainieri, A. Renzini, M. Scodeggio, S. Bardelli, M. Brusa, K. Caputi, F. Civano, G. Coppa, O. Cucciati, S. de la Torre, L. de Ravel, P. Franzetti, B. Garilli, C. Halliday, G. Hasinger, A. M. Koekemoer, A. Iovino, P. Kampczyk, C. Knobel, K. Kovač, J.-F. Le Borgne, V. Le Brun, C. Maier, A. Merloni, P. Nair, R. Pello, Y. Peng, E. P. Montero, E. Ricciardelli, M. Salvato, J. Silverman, M. Tanaka, L. Tasca, L. Tresse, D. Vergani, E. Zucca, U. Abbas, D. Bottini, A. Cappi, P. Cassata, A. Cimatti, L. Guzzo, A. Leauthaud, D. Maccagni, C. Marinoni, H. J. M. Cracken, P. Memeo, B. Meneux, P. Oesch, C. Porciani, L. Pozzetti, R. Scaramella, The [O

III] emission line luminosity function of optically selected type-2 AGN from zCOSMOS. *Astron. Astrophys.* **510**, A56 (2010).

99. D. C. Leonard, A. V. Filippenko, Spectropolarimetry of the type II supernovae 1997ds, 1998A, and 1999gi. *Publ. Astron. Soc. Pac.* **113**, 920–936 (2001).

100. HI4PI Collaboration, N. Ben Bekhti, L. Flöer, R. Keller, J. Kerp, D. Lenz, B. Winkel, J. Bailin, M. R. Calabretta, L. Dedes, H. A. Ford, B. K. Gibson, U. Haud, S. Janowiecki, P. M. W. Kalberla, F. J. Lockman, N. M. McClure-Griffiths, T. Murphy, H. Nakanishi, D. J. Pisano, L. Staveley-Smith, HI4PI: A full-sky H i survey based on EBHIS and GASS. *Astron. Astrophys.* **594**, A116 (2016).

101. M. Gilfanov, Low-mass X-ray binaries as a stellar mass indicator for the host galaxy. *Mon. Not. R. Astron. Soc.* **349**, 146–168 (2004).

102. G. Riccio, G. Yang, K. Małek, M. Boquien, F. P. Junais, M. Hamed, M. Grespan, M. Paolillo, O. Torbaniuk, X-ray luminosity-star formation rate scaling relation: Constraints from the eROSITA Final Equatorial Depth Survey (eFEDS). *Astron. Astrophys.* **678**, A164 (2023).

103. M. M. Kasliwal, S. R. Kulkarni, A. Gal-Yam, P. E. Nugent, M. Sullivan, L. Bildsten, O. Yaron, H. B. Perets, I. Arcavi, S. Ben-Ami, V. B. Bhalerao, J. S. Bloom, S. B. Cenko, A. V. Filippenko, D. A. Frail, M. Ganeshalingam, A. Horesh, D. A. Howell, N. M. Law, D. C. Leonard, W. Li, E. O. Ofek, D. Polishook, D. Poznanski, R. M. Quimby, J. M. Silverman, A. Sternberg, D. Xu, Calcium-rich gap transients in the remote outskirts of galaxies. *Astrophys. J.* **755**, 161 (2012).

104. A. Gal-Yam, The most luminous supernovae. *Ann. Rev. Astron. Astrophys.* **57**, 305–333 (2019).

105. E. Berger, A. M. Soderberg, R. A. Chevalier, C. Fransson, R. J. Foley, D. C. Leonard, J. H. Debes, A. M. Diamond-Stanic, A. K. Dupree, I. I. Ivans, J. Simmerer, I. B. Thompson, C. A. Tremonti, An intermediate luminosity transient in NGC 300: The eruption of a dust-enshrouded massive star. *Astrophys. J.* **699**, 1850–1865 (2009).

106. A. Pastorello, E. Mason, S. Taubenberger, M. Fraser, G. Cortini, L. Tomasella, M. T. Botticella, N. Elias-Rosa, R. Kotak, S. J. Smartt, S. Benetti, E. Cappellaro, M. Turatto, L. Tartaglia, S. G. Djorgovski, A. J. Drake, M. Berton, F. Briganti, J. Brimacombe, F. Bufano, Y.-Z. Cai, S. Chen, E. J. Christensen, F. Ciabattari, E. Congiu, A. Dimai, C. Inserra, E. Kankare, L. Magill, K. Maguire, F. Martinelli, A. Morales-Garoffolo, P. Ochner, G. Pignata, A. Reguitti, J. Sollerman, S. Spiro, G. Terreran, D. E. Wright, Luminous red novae: Stellar mergers or giant eruptions? *Astron. Astrophys.* **630**, A75 (2019).

107. J. T. Hinkle, Mid-infrared echoes of ambiguous nuclear transients reveal high dust covering fractions: Evidence for dusty tori. *Mon. Not. R. Astron. Soc.* **531**, 2603–2614 (2024).

108. J. T. Hinkle, C. S. Kochanek, B. J. Shappee, P. J. Vallely, K. Auchettl, M. Fausnaugh, T. W.-S. Holoien, H. P. Treiber, A. V. Payne, B. S. Gaudi, K. G. Stassun, T. A. Thompson, J. L. Tonry, S. Villanueva Jr., TESS shines light on the origin of the ambiguous nuclear transient ASASSN-18el. *Mon. Not. R. Astron. Soc.* **521**, 3517–3526 (2023).

109. P. Wiseman, R. D. Williams, I. Arcavi, L. Galbany, M. J. Graham, S. Hönig, M. Newsome, B. Subrayan, M. Sullivan, Y. Wang, D. Ilić, M. Nicholl, S. Oates, T. Petrushevska, K. W. Smith, A systematically selected sample of luminous, long-duration, ambiguous nuclear transients. *Mon. Not. R. Astron. Soc.* **537**, 2024–2045 (2025).

110. J. T. Hinkle, B. J. Shappee, M. A. Tucker, A swift fix II: Physical parameters of type I superluminous supernovae. arXiv:2309.03270 (2023).

111. D. Godoy-Rivera, K. Z. Stanek, C. S. Kochanek, P. Chen, S. Dong, J. L. Prieto, B. J. Shappee, S. W. Jha, R. J. Foley, Y.-C. Pan, T. W.-S. Holoien, T. A. Thompson, D. Grupe, J. F. Beacom, The unexpected, long-lasting, UV rebrightening of the superluminous supernova ASASSN-15lh. *Mon. Not. R. Astron. Soc.* **466**, 1428–1443 (2017).

112. M. Nicholl, S. J. Smartt, A. Jerkstrand, C. Inserra, S. A. Sim, T.-W. Chen, S. Benetti, M. Fraser, A. Gal-Yam, E. Kankare, K. Maguire, K. Smith, M. Sullivan, S. Valenti, D. R. Young, C. Baltay, F. E. Bauer, S. Baumont, D. Bersier, M.-T. Botticella, M. Childress, M. Dennefeld, M. D. Valle, N. Elias-Rosa, U. Feindt, L. Galbany, E. Hadjiyska, L. Le Guillou, G. Leloudas, P. Mazzali, R. M. Kinnon, J. Polshaw, D. Rabinowitz, S. Rostami, R. Scalzo,

B. P. Schmidt, S. Schulze, J. Sollerman, F. Taddia, F. Yuan, On the diversity of superluminous supernovae: Ejected mass as the dominant factor. *Mon. Not. R. Astron. Soc.* **452**, 3869–3893 (2015).

113. A. Savitzky, M. J. E. Golay, Smoothing and differentiation of data by simplified least squares procedures. *Anal. Chem.* **36**, 1627–1639 (1964).

114. S. Vaughan, R. Edelson, R. S. Warwick, P. Uttley, On characterizing the variability properties of X-ray light curves from active galaxies. *Mon. Not. R Astron. Soc.* **345**, 1271–1284 (2003).

115. N. Jiang, T. Wang, X. Hu, L. Sun, L. Dou, L. Xiao, Infrared echoes of optical tidal disruption events: ~1% dust-covering factor or less at subparsec scale. *Astrophys. J.* **911**, 31 (2021).

116. S. B. Cenko, A. Cucchiara, N. Roth, S. Veilleux, J. X. Prochaska, L. Yan, J. Guillochon, W. P. Maksym, I. Arcavi, N. R. Butler, A. V. Filippenko, A. S. Fruchter, S. Gezari, D. Kasen, A. J. Levan, J. M. Miller, D. R. Pasham, E. Ramirez-Ruiz, L. E. Strubbe, N. R. Tanvir, F. Tombesi, An ultraviolet spectrum of the tidal disruption flare ASASSN-14li. *Astrophys. J. Lett.* **818**, L32 (2016).

117. J. S. Brown, C. S. Kochanek, T. W.-S. Holoien, K. Z. Stanek, K. Auchettl, B. J. Shappee, J. L. Prieto, N. Morrell, E. Falco, J. Strader, L. Chomiuk, R. Post, S. Villanueva Jr., S. Mathur, S. Dong, P. Chen, S. Bose, The ultraviolet spectroscopic evolution of the low-luminosity tidal disruption event iPTF16fnl. *Mon. Not. R. Astron. Soc.* **473**, 1130–1144 (2018).

118. T. Hung, R. J. Foley, S. Veilleux, S. B. Cenko, J. L. Dai, K. Auchettl, T. G. Brink, G. Dimitriadis, A. V. Filippenko, S. Gezari, T. W.-S. Holoien, C. D. Kilpatrick, B. Mockler, A. L. Piro, E. Ramirez-Ruiz, C. Rojas-Bravo, M. R. Siebert, S. van Velzen, W. K. Zheng, Discovery of a fast iron low-ionization outflow in the early evolution of the nearby tidal disruption event AT 2019qiz. *Astrophys. J.* **917**, 9 (2021).

119. S. A. Grandi, M. M. Phillips, Mg II lambda 2798 emission in QSOs, Seyfert 1 galaxies, and broad-line radio galaxies. *Astrophys. J.* **232**, 659–669 (1979).

120. R. J. McLure, J. S. Dunlop, The cosmological evolution of quasar black hole masses. *Mon. Not. R. Astron. Soc.* **352**, 1390–1404 (2004).

121. J.-G. Wang, X.-B. Dong, T.-G. Wang, L. C. Ho, W. Yuan, H. Wang, K. Zhang, S. Zhang, H. Zhou, Estimating black hole masses in active galactic nuclei using the Mg II λ2800 emission line. *Astrophys. J.* **707**, 1334–1346 (2009).

122. X.-B. Dong, T.-G. Wang, J.-G. Wang, X. Fan, H. Wang, H. Zhou, W. Yuan, Eddington ratio governs the equivalent width of Mg II emission line in active galactic nuclei. *Astrophys. J.* **703**, L1–L5 (2009).

123. Y. Shen, G. T. Richards, M. A. Strauss, P. B. Hall, D. P. Schneider, S. Snedden, D. Bizyaev, H. Brewington, V. Malanushenko, E. Malanushenko, D. Oravetz, K. Pan, A. Simmons, A catalog of quasar properties from sloan digital sky survey data Release 7. *Astrophys. J. Suppl. Ser.* **194**, 45 (2011).

124. H. A. N. Le, J.-H. Woo, Y. Xue, Calibrating Mg II-based black hole mass estimators using low-to-high-luminosity active galactic nuclei. *Astrophys. J.* **901**, 35 (2020).

125. H.-Y. Liu, W.-J. Liu, X.-B. Dong, H. Zhou, T. Wang, H. Lu, W. Yuan, A comprehensive and uniform sample of broad-line active galactic nuclei from the SDSS DR7. *Astrophys. J. Suppl. Ser.* **243**, 21 (2019).

126. P. Charalampopoulos, G. Leloudas, D. B. Malesani, T. Wevers, I. Arcavi, M. Nicholl, M. Pursiainen, A. Lawrence, J. P. Anderson, S. Benetti, G. Cannizzaro, T.-W. Chen, L. Galbany, M. Gromadzki, C. P. Gutiérrez, C. Inserra, P. G. Jonker, T. E. Müller-Bravo, F. Onori, P. Short, J. Sollerman, D. R. Young, A detailed spectroscopic study of tidal disruption events. *Astron. Astrophys.* **659**, A34 (2022).

127. F. Taddia, M. D. Stritzinger, J. Sollerman, M. M. Phillips, J. P. Anderson, L. Boldt, A. Campillay, S. Castellón, C. Contreras, G. Folatelli, M. Hamuy, E. Heinrich-Josties, W. Krzeminski, N. Morrell, C. R. Burns, W. L. Freedman, B. F. Madore, S. E. Persson, N. B. Suntzeff, Carnegie Supernova Project: Observations of type IIn supernovae. *Astron. Astrophys.* **555**, A10 (2013).

128. F. Jansen, D. Lumb, B. Altieri, J. Clavel, M. Ehle, C. Erd, C. Gabriel, M. Guainazzi, P. Gondoin, R. Much, R. Munoz, M. Santos, N. Schartel, D. Texier, G. Vacanti, XMM-Newton observatory. I. The spacecraft and operations. *Astron. Astrophys.* **365**, L1–L6 (2001).

129. M. C. Weisskopf, B. Brinkman, C. Canizares, G. Garmire, S. Murray, L. P. Van Speybroeck, An overview of the performance and scientific results from the chandra x-ray observatory. *Publ. Astron. Soc. Pac.* **114**, 1–24 (2002).

130. K. Auchettl, E. Ramirez-Ruiz, J. Guillochon, A comparison of the x-ray emission from tidal disruption events with those of active galactic nuclei. *Astrophys. J.* **852**, 37 (2018).

131. J. S. Brown, T. W.-S. Holoien, K. Auchettl, K. Z. Stanek, C. S. Kochanek, B. J. Shappee, J. L. Prieto, D. Grupe, The long term evolution of ASASSN-14li. *Mon. Not. R. Astron. Soc.* **466**, 4904–4916 (2017).

132. T. W.-S. Holoien, J. S. Brown, K. Auchettl, C. S. Kochanek, J. L. Prieto, B. J. Shappee, J. Van Saders, The unusual late-time evolution of the tidal disruption event ASASSN-15oi. *Mon. Not. R. Astron. Soc.* **480**, 5689–5703 (2018).

133. T. Wevers, D. R. Pasham, S. van Velzen, G. Leloudas, S. Schulze, J. C. A. Miller-Jones, P. G. Jonker, M. Gromadzki, E. Kankare, S. T. Hodgkin, Ł. Wyrzykowski, Z. Kostrzewa-Rutkowska, S. Moran, M. Berton, K. Maguire, F. Onori, S. Mattila, M. Nicholl, Evidence for rapid disc formation and reprocessing in the X-ray bright tidal disruption event candidate AT 2018fyk. *Mon. Not. R. Astron. Soc.* **488**, 4816–4830 (2019).

134. J. T. Hinkle, T. W.-S. Holoien, K. Auchettl, B. J. Shappee, J. M. M. Neustadt, A. V. Payne, J. S. Brown, C. S. Kochanek, K. Z. Stanek, M. J. Graham, M. A. Tucker, A. Do, J. P. Anderson, S. Bose, P. Chen, D. A. Coulter, G. Dimitriadis, S. Dong, R. J. Foley, M. E. Huber, T. Hung, C. D. Kilpatrick, G. Pignata, A. L. Piro, C. Rojas-Bravo, M. R. Siebert, B. Stalder, T. A. Thompson, J. L. Tonry, P. J. Vallely, J. P. Wisniewski, Discovery and follow-up of ASASSN-19dj: An X-ray and UV luminous TDE in an extreme post-starburst galaxy. *Mon. Not. R. Astron. Soc.* **500**, 1673–1696 (2021).

135. B. Trakhtenbrot, I. Arcavi, C. L. MacLeod, C. Ricci, E. Kara, M. L. Graham, D. Stern, F. A. Harrison, J. Burke, D. Hiramatsu, G. Hosseinzadeh, D. A. Howell, S. J. Smartt, A. Rest, J. L. Prieto, B. J. Shappee, T. W.-S. Holoien, D. Bersier, A. V. Filippenko, T. G. Brink, W. K. Zheng, R. Li, R. A. Remillard, M. Loewenstein, 1ES 1927+654: An AGN caught changing look on a timescale of months. *Astrophys. J.* **883**, 94 (2019).

136. D. W. Just, W. N. Brandt, O. Shemmer, A. T. Steffen, D. P. Schneider, G. Chartas, G. P. Garmire, The X-ray properties of the most luminous quasars from the sloan digital sky survey. *Astrophys. J.* **665**, 1004–1022 (2007).

137. C. Auge, D. Sanders, E. Treister, C. M. Urry, A. Kirkpatrick, N. Cappelluti, T. T. Ananna, M. Boquien, M. Baloković, F. Civano, B. Coleman, A. Ghosh, J. Kartaltepe, M. Koss, S. LaMassa, S. Marchesi, A. Peca, M. Powell, B. Trakhtenbrot, T. J. Turner, The accretion history of AGN: The spectral energy distributions of X-ray-luminous active galactic nuclei. *Astrophys. J.* **957**, 19 (2023).

138. T. Wevers, S. van Velzen, P. G. Jonker, N. C. Stone, T. Hung, F. Onori, S. Gezari, N. Blagorodnova, Black hole masses of tidal disruption event host galaxies. *Mon. Not. R. Astron. Soc.* **471**, 1694–1708 (2017).

139. M. Schmidt, Space distribution and luminosity functions of quasi-stellar radio sources. *Astrophys. J.* **151**, 393 (1968).

140. N. Gehrels, Confidence limits for small numbers of events in astrophysical data. *Astrophys. J.* **303**, 336 (1986).

141. S. van Velzen, G. R. Farrar, Measurement of the rate of stellar tidal disruption flares. *Astrophys. J.* **792**, 53 (2014).

142. Y. Yao, V. Ravi, S. Gezari, S. van Velzen, W. Lu, S. Schulze, J. J. Somalwar, S. R. Kulkarni, E. Hammerstein, M. Nicholl, M. J. Graham, D. A. Perley, S. B. Cenko, R. Stein, A. Ricarte, U. Chadayammuri, E. Quataert, E. C. Bellm, J. S. Bloom, R. Dekany, A. J. Drake, S. L. Groom, A. A. Mahabal, T. A. Prince, R. Riddle, B. Rusholme, Y. Sharma, J. Sollerman, L. Yan, Tidal disruption event demographics with the Zwicky transient facility: Volumetric

rates, luminosity function, and implications for the local black hole mass function. *Astrophys. J. Lett.* **955**, L6 (2023).

143. R. M. Quimby, F. Yuan, C. Akerlof, J. C. Wheeler, Rates of superluminous supernovae at z □ 0.2. *Mon. Not. R. Astron. Soc.* **431**, 912–922 (2013).

144. W.-C. Zhao, X.-X. Xue, X.-F. Cao, On the event rate and luminosity function of superluminous supernovae. *New Astron.* **83**, 101506 (2021).

145. D. D. Desai, C. S. Kochanek, B. J. Shappee, T. Jayasinghe, K. Z. Stanek, T. W.-S. Holoien, T. A. Thompson, C. Ashall, J. F. Beacom, A. Do, S. Dong, J. L. Prieto, Supernova rates and luminosity functions from ASAS-SN I: 2014–2017 Type Ia SNe and their subtypes. *Mon. Not. R. Astron. Soc.* **530**, 5016–5029 (2024).

146. C. S. Kochanek, The flat-spectrum radio luminosity function, gravitational lensing, galaxy ellipticities, and cosmology. *Astrophys. J.* **473**, 595 (1996).

147. A. Georgakakis, K. Nandra, E. S. Laird, J. Aird, M. Trichas, A new method for determining the sensitivity of X-ray imaging observations and the X-ray number counts. *Mon. Not. R. Astron. Soc.* **388**, 1205–1213 (2008).

148. B. D. Lehmer, Y. Q. Xue, W. N. Brandt, D. M. Alexander, F. E. Bauer, M. Brusa, A. Comastri, R. Gilli, A. E. Hornschemeier, B. Luo, M. Paolillo, A. Ptak, O. Shemmer, D. P. Schneider, P. Tozzi, C. Vignali, The 4 Ms Chandra deep field-south number counts apportioned by source class: Pervasive active galactic nuclei and the ascent of normal galaxies. *Astrophys. J.* **752**, 46 (2012).

149. S. M. Faber, R. E. Jackson, Velocity dispersions and mass-to-light ratios for elliptical galaxies. *Astrophys. J.* **204**, 668–683 (1976).

150. D. K. Nadyozhin, The properties of NI CO Fe decay. *Astrophys. J.* **92**, 527 (1994).

151. R. M. Quimby, S. R. Kulkarni, M. M. Kasliwal, A. Gal-Yam, I. Arcavi, M. Sullivan, P. Nugent, R. Thomas, D. A. Howell, E. Nakar, L. Bildsten, C. Theissen, N. M. Law, R. Dekany, G. Rahmer, D. Hale, R. Smith, E. O. Ofek, J. Zolkower, V. Velur, R. Walters, J.

Henning, K. Bui, D. M. Kenna, D. Poznanski, S. B. Cenko, D. Levitan, Hydrogen-poor superluminous stellar explosions. *Nature* **474**, 487–489 (2011).

152. J. P. Ostriker, J. E. Gunn, Do pulsars make supernovae? *Astrophys. J.* **164**, L95 (1971).

153. R. W. Romani, D. Kandel, A. V. Filippenko, T. G. Brink, W. Zheng, PSR J0952-0607: The fastest and heaviest known galactic neutron star. *Astrophys. J. Lett.* **934**, L17 (2022).

154. M.-A. Hashimoto, K. Oyamatsu, Y. Eriguchi, Upper limit of the angular velocity of neutron stars. *Astrophys. J.* **436**, 257 (1994).

155. G. F. Burgio, H. J. Schulze, F. Weber, On the maximum rotational frequency of neutron and hybrid stars. *Astron. Astrophys.* **408**, 675–680 (2003).

156. P. Kaaret, Z. Prieskorn, J. J. M. i. 't Zand, S. Brandt, N. Lund, S. Mereghetti, D. Götz, E. Kuulkers, J. A. Tomsick, Evidence of 1122 Hz X-ray burst oscillations from the neutron star X-ray transient XTE J1739-285. *Astrophys. J.* **657**, L97–L100 (2007).

157. C. Inserra, S. J. Smartt, E. E. E. Gall, G. Leloudas, T.-W. Chen, S. Schulze, A. Jerkstrand, M. Nicholl, J. P. Anderson, I. Arcavi, S. Benetti, R. A. Cartier, M. Childress, M. Della Valle, H. Flewelling, M. Fraser, A. Gal-Yam, C. P. Gutiérrez, G. Hosseinzadeh, D. A. Howell, M. Huber, E. Kankare, T. Krühler, E. A. Magnier, K. Maguire, C. McCully, S. Prajs, N. Primak, R. Scalzo, B. P. Schmidt, M. Smith, K. W. Smith, B. E. Tucker, S. Valenti, M. Wilman, D. R. Young, F. Yuan, On the nature of hydrogen-rich superluminous supernovae. *Mon. Not. R. Astron. Soc.* **475**, 1046–1072 (2018).

158. Y. Ueda, M. Akiyama, G. Hasinger, T. Miyaji, M. G. Watson, Toward the standard population synthesis model of the X-ray background: Evolution of X-ray luminosity and absorption functions of active galactic nuclei including compton-thick populations. *Astrophys. J.* **786**, 104 (2014).

159. M. Giustini, D. Proga, A global view of the inner accretion and ejection flow around super massive black holes. Radiation-driven accretion disk winds in a physical context. *Astron. Astrophys.* **630**, A94 (2019).

160. J. P. Dunn, D. M. Crenshaw, S. B. Kraemer, J. R. Gabel, A survey of intrinsic absorption in active galaxies using the far ultraviolet spectroscopic explorer. *Astron. J.* **134**, 1061–1071 (2007).

161. T. J. Moriya, K. Maeda, F. Taddia, J. Sollerman, S. I. Blinnikov, E. I. Sorokina, An analytic bolometric light curve model of interaction-powered supernovae and its application to Type IIn supernovae. *Mon. Not. R. Astron. Soc.* **435**, 1520–1535 (2013).

162. D. Tsuna, K. Kashiyama, T. Shigeyama, Type IIn supernova light curves powered by forward and reverse shocks. *Astrophys. J.* **884**, 87 (2019).

163. F. Honma, R. Matsumoto, S. Kato, Nonlinear oscillations of thermally unstable slim accretion disks around a neutron star or a black hole. *Publ. Astron. Soc. Jpn.* **43**, 147–168 (1991).

164. K. Ohsuga, Two-dimensional radiation-hydrodynamic model for limit-cycle oscillations of luminous accretion disks. *Astrophys. J.* **640**, 923–928 (2006).

165. A. Janiuk, B. Czerny, On different types of instabilities in black hole accretion discs: Implications for X-ray binaries and active galactic nuclei. *Mon. Not. R. Astron. Soc.* **414**, 2186–2194 (2011).

166. G. V. Lipunova, A. S. Tavleev, K. L. Malanchev, Fast giant flares in discs around supermassive black holes. arXiv:2404.08441 (2024).

167. F. Yuan, R. Narayan, Hot accretion flows around black holes. *Annu. Rev. Astron. Astrophys.* **52**, 529–588 (2014).

168. E. Hammerstein, S. van Velzen, S. Gezari, S. B. Cenko, Y. Yao, C. Ward, S. Frederick, N. Villanueva, J. J. Somalwar, M. J. Graham, S. R. Kulkarni, D. Stern, I. Andreoni, E. C. Bellm, R. Dekany, S. Dhawan, A. J. Drake, C. Fremling, P. Gatkine, S. L. Groom, A. Y. Q. Ho, M. M. Kasliwal, V. Karambelkar, E. C. Kool, F. J. Masci, M. S. Medford, D. A. Perley, J. Purdum, J. van Roestel, Y. Sharma, J. Sollerman, K. Taggart, L. Yan, The final season reimagined: 30 tidal disruption events from the ZTF-I survey. *Astrophys. J.* **942**, 9 (2023).

169. O. Demircan, G. Kahraman, Stellar mass-luminosity and mass-radius relations. *Astrophys. Space Sci.* **181**, 313–322 (1991).

170. C. A. Tout, O. R. Pols, P. P. Eggleton, Z. Han, Zero-age main-sequence radii and luminosities as analytic functions of mass and metallicity. *Mon. Not. R. Astron. Soc.* **281**, 257–262 (1996).

171. J. G. Hills, Possible power source of Seyfert galaxies and QSOs. *Nature* **254**, 295–298 (1975).

172. R. K. Chowdhury, J. N. Y. Chang, L. Dai, P. Natarajan, Detecting population III stars through tidal disruption events in the era of JWST and Roman. *Astrophys. J. Lett.* **966**, L33 (2024).

173. A. Bandopadhyay, J. Fancher, A. Athian, V. Indelicato, S. Kapalanga, A. Kumah, D. A. Paradiso, M. Todd, E. R. Coughlin, C. J. Nixon, The peak of the fallback rate from tidal disruption events: Dependence on stellar type. *Astrophys. J. Lett.* **961**, L2 (2024).

174. P. Kroupa, On the variation of the initial mass function. *Mon. Not. R. Astron. Soc.* **322**, 231–246 (2001).

175. G. Chabrier, Galactic stellar and substellar initial mass function. *Publ. Astron. Soc. Pac.* **115**, 763–795 (2003).

176. H. Maness, F. Martins, S. Trippe, R. Genzel, J. R. Graham, C. Sheehy, M. Salaris, S. Gillessen, T. Alexander, T. Paumard, T. Ott, R. Abuter, F. Eisenhaue, Evidence for a long-standing top-heavy initial mass function in the central parsec of the galaxy. *Astrophys. J.* **669**, 1024–1041 (2007).

177. H. Bartko, F. Martins, S. Trippe, T. K. Fritz, R. Genzel, T. Ott, F. Eisenhauer, S. Gillessen, T. Paumard, T. Alexander, K. Dodds-Eden, O. Gerhard, Y. Levin, L. Mascetti, S. Nayakshin, H. B. Perets, G. Perrin, O. Pfuhl, M. J. Reid, D. Rouan, M. Zilka, A. Sternberg, An extremely top-heavy initial mass function in the galactic center stellar disks. *Astrophys. J.* **708**, 834–840 (2010).

178. D. E. Vanden Berk, G. T. Richards, A. Bauer, M. A. Strauss, D. P. Schneider, T. M. Heckman, D. G. York, P. B. Hall, X. Fan, G. R. Knapp, S. F. Anderson, J. Annis, N. A. Bahcall, M. Bernardi, J. W. Briggs, J. Brinkmann, R. Brunner, S. Burles, L. Carey, F. J. Castander, A. J. Connolly, J. H. Crocker, I. Csabai, M. Doi, D. Finkbeiner, S. Friedman, J. A. Frieman, M. Fukugita, J. E. Gunn, G. S. Hennessy, Ž. Ivezić, S. Kent, P. Z. Kunszt, D. Q. Lamb, R. F. Leger, D. C. Long, J. Loveday, R. H. Lupton, A. Meiksin, A. Merelli, J. A. Munn, H. J. Newberg, M. Newcomb, R. C. Nichol, R. Owen, J. R. Pier, A. Pope, C. M. Rockosi, D. J. Schlegel, W. A. Siegmund, S. Smee, Y. Snir, C. Stoughton, C. Stubbs, M. S. Rao, A. S. Szalay, G. P. Szokoly, C. Tremonti, A. Uomoto, P. Waddell, B. Yanny, W. Zheng, Composite quasar spectra from the Sloan digital sky survey. *Astron. J.* **122**, 549–564 (2001).

179. C. S. Kochanek, B. J. Shappee, K. Z. Stanek, T. W.-S. Holoien, T. A. Thompson, J. L. Prieto, S. Dong, J. V. Shields, D. Will, C. Britt, D. Perzanowski, G. Pojmański, The All-Sky Automated Survey for Supernovae (ASAS-SN) light curve server v1.0. *Publ. Astron. Soc. Pac.* **129**, 104502 (2017).

180. K. Hart, B. J. Shappee, D. Hey, C. S. Kochanek, K. Z. Stanek, L. Lim, S. Dobbs, M. Tucker, T. Jayasinghe, J. F. Beacom, T. Boright, T. Holoien, J. M. Joel Ong, J. L. Prieto, T. A. Thompson, D. Will, ASAS-SN Sky Patrol V2.0. arXiv:2304.03791 (2023).

181. E. C. Bellm, S. R. Kulkarni, M. J. Graham, R. Dekany, R. M. Smith, R. Riddle, F. J. Masci, G. Helou, T. A. Prince, S. M. Adams, C. Barbarino, T. Barlow, J. Bauer, R. Beck, J. Belicki, R. Biswas, N. Blagorodnova, D. Bodewits, B. Bolin, V. Brinnel, T. Brooke, B. Bue, M. Bulla, R. Burruss, S. B. Cenko, C.-K. Chang, A. Connolly, M. Coughlin, J. Cromer, V. Cunningham, K. De, A. Delacroix, V. Desai, D. A. Duev, G. Eadie, T. L. Farnham, M. Feeney, U. Feindt, D. Flynn, A. Franckowiak, S. Frederick, C. Fremling, A. Gal-Yam, S. Gezari, M. Giomi, D. A. Goldstein, V. Z. Golkhou, A. Goobar, S. Groom, E. Hacopians, D. Hale, J. Henning, A. Y. Q. Ho, D. Hover, J. Howell, T. Hung, D. Huppenkothen, D. Imel, W.-H. Ip, Ž. Ivezić, E. Jackson, L. Jones, M. Juric, M. M. Kasliwal, S. Kaspi, S. Kaye, M. S. P. Kelley, M. Kowalski, E. Kramer, T. Kupfer, W. Landry, R. R. Laher, C.-D. Lee, H. W. Lin, Z.-Y. Lin, R. Lunnan, M. Giomi, A. Mahabal, P. Mao, A. A. Miller, S. Monkewitz, P. Murphy, C.-C. Ngeow, J. Nordin, P. Nugent, E. Ofek, M. T. Patterson, B. Penprase, M. Porter, L. Rauch, U. Rebbapragada, D. Reiley, M. Rigault, H. Rodriguez, J. van Roestel, B.

Rusholme, J. van Santen, S. Schulze, D. L. Shupe, L. P. Singer, M. T. Soumagnac, R. Stein, J. Surace, J. Sollerman, P. Szkody, F. Taddia, S. Terek, A. Van Sistine, S. van Velzen, W. T. Vestrand, R. Walters, C. Ward, Q.-Z. Ye, P.-C. Yu, L. Yan, J. Zolkower, The Zwicky transient facility: System overview, performance, and first results. *Publ. Astron. Soc. Pac.* **131**, 018002 (2019).

182. F. J. Masci, R. R. Laher, B. Rusholme, D. L. Shupe, S. Groom, J. Surace, E. Jackson, S. Monkewitz, R. Beck, D. Flynn, S. Terek, W. Landry, E. Hacopians, V. Desai, J. Howell, T. Brooke, D. Imel, S. Wachter, Q.-Z. Ye, H.-W. Lin, S. B. Cenko, V. Cunningham, U. Rebbapragada, B. Bue, A. A. Miller, A. Mahabal, E. C. Bellm, M. T. Patterson, M. Jurić, V. Z. Golkhou, E. O. Ofek, R. Walters, M. Graham, M. M. Kasliwal, R. G. Dekany, T. Kupfer, K. Burdge, C. B. Cannella, T. Barlow, A. Van Sistine, M. Giomi, C. Fremling, N. Blagorodnova, D. Levitan, R. Riddle, R. M. Smith, G. Helou, T. A. Prince, S. R. Kulkarni, The Zwicky transient facility: Data processing, products, and archive. *Publ. Astron. Soc. Pac.* **131**, 018003 (2019).

183. M. A. Tucker, B. J. Shappee, M. E. Huber, A. V. Payne, A. Do, J. T. Hinkle, T. de Jaeger, C. Ashall, D. D. Desai, W. B. Hoogendam, G. Aldering, K. Auchettl, C. Baranec, J. Bulger, K. Chambers, M. Chun, K. W. Hodapp, T. B. Lowe, L. McKay, R. Rampy, D. Rubin, J. L. Tonry, The Spectroscopic Classification of Astronomical Transients (SCAT) survey: Overview, pipeline description, initial results, and future plans. *Publ. Astron. Soc. Pac.* **134**, 124502 (2022).

184. F. Acero, M. Ackermann, M. Ajello, A. Albert, W. B. Atwood, M. Axelsson, L. Baldini, J. Ballet, G. Barbiellini, D. Bastieri, A. Belfiore, R. Bellazzini, E. Bissaldi, R. D. Blandford, E. D. Bloom, J. R. Bogart, R. Bonino, E. Bottacini, J. Bregeon, R. J. Britto, P. Bruel, R. Buehler, T. H. Burnett, S. Buson, G. A. Caliandro, R. A. Cameron, R. Caputo, M. Caragiulo, P. A. Caraveo, J. M. Casandjian, E. Cavazzuti, E. Charles, R. C. G. Chaves, A. Chekhtman, C. C. Cheung, J. Chiang, G. Chiaro, S. Ciprini, R. Claus, J. Cohen- Tanugi, L. R. Cominsky, J. Conrad, S. Cutini, F. D'Ammando, A. de Angelis, M. De Klotz, F. de Palma, R. Desiante, S. W. Digel, L. Di Venere, P. S. Drell, R. Dubois, D. Dumora, C. Favuzzi, S. J. Fegan, E. C. Ferrara, J. Finke, A. Franckowiak, Y. Fukazawa, S. Funk, P. Fusco, F. Gargano, D. Gasparrini, B. Giebels, N. Giglietto, P. Giommi, F. Giordano, M. Giroletti, T. Glanzman, G.

Godfrey, I. A. Grenier, M.-H. Grondin, J. E. Grove, L. Guillemot, S. Guiriec, D. Hadasch, A. K. Harding, E. Hays, J. W. Hewitt, A. B. Hill, D. Horan, G. Iafrate, T. Jogler, G. Jóhannesson, R. P. Johnson, A. S. Johnson, T. J. Johnson, W. N. Johnson, T. Kamae, J. Kataoka, J. Katsuta, M. Kuss, G. La Mura, D. Landriu, S. Larsson, L. Latronico, M. Lemoine- Goumard, J. Li, L. Li, F. Longo, F. Loparco, B. Lott, M. N. Lovellette, P. Lubrano, G. M. Madejski, F. Massaro, M. Mayer, M. N. Mazziotta, J. E. McEnery, P. F. Michelson, N. Mirabal, T. Mizuno, A. A. Moiseev, M. Mongelli, M. E. Monzani, A. Morselli, I. V. Moskalenko, S. Murgia, E. Nuss, M. Ohno, T. Ohsugi, N. Omodei, M. Orienti, E. Orlando, J. F. Ormes, D. Paneque, J. H. Panetta, J. S. Perkins, M. Pesce- Rollins, F. Piron, G. Pivato, T. A. Porter, J. L. Racusin, R. Rando, M. Razzano, S. Razzaque, A. Reimer, O. Reimer, T. Reposeur, L. S. Rochester, R. W. Romani, D. Salvetti, M. Sánchez-Conde, P. M. S. Parkinson, A. Schulz, E. J. Siskind, D. A. Smith, F. Spada, G. Spandre, P. Spinelli, T. E. Stephens, A. W. Strong, D. J. Suson, H. Takahashi, T. Takahashi, Y. Tanaka, J. G. Thayer, J. B. Thayer, D. J. Thompson, L. Tibaldo, O. Tibolla, D. F. Torres, E. Torresi, G. Tosti, E. Troja, B. Van Klaveren, G. Vianello, B. L. Winer, K. S. Wood, M. Wood, S. Zimmer, Fermi large area telescope third source catalog. *Astrophys. J. Suppl. Ser.* **218**, 23 (2015).

185. F. Ochsenbein, P. Bauer, J. Marcout, The VizieR database of astronomical catalogues. *Astron. Astrophys. Suppl. Ser.* **143**, 23–32 (2000).

186. A. Delgado, D. Harrison, S. Hodgkin, M. V. Leeuwen, G. Rixon, A. Yoldas, "GaiaAlerts Transient Discovery Report for 2016-07-21," in *Transient Name Server Discovery Report 2016-480* (2016).

187. A. Delgado, D. Harrison, S. Hodgkin, M. V. Leeuwen, G. Rixon, A. Yoldas, "GaiaAlerts Transient Discovery Report for 2018-08-13," in *Transient Name Server Discovery Report 2018-1165* (2018).

188. J. Nordin, V. Brinnel, J. V. Santen, A. Gal-Yam, O. Yaron, S. Schulze, "AMPEL/ZTF Transient Discovery Report for 2021-05-10," in *Transient Name Server Discovery Report 2021-1548* (2021), pp. 1–1548.

189. T. Robitaille, E. Bressert, APLpy: Astronomical Plotting Library in Python (Astrophysics Source Code Library, record ascl:1208.017, 2012).

190. K. C. Chambers, E. A. Magnier, N. Metcalfe, H. A. Flewelling, M. E. Huber, C. Z. Waters, L. Denneau, P. W. Draper, D. Farrow, D. P. Finkbeiner, C. Holmberg, J. Koppenhoefer, P. A. Price, A. Rest, R. P. Saglia, E. F. Schlafly, S. J. Smartt, W. Sweeney, R. J. Wainscoat, W. S. Burgett, S. Chastel, T. Grav, J. N. Heasley, K. W. Hodapp, R. Jedicke, N. Kaiser, R.-P. Kudritzki, G. A. Luppino, R. H. Lupton, D. G. Monet, J. S. Morgan, P. M. Onaka, B. Shiao, C. W. Stubbs, J. L. Tonry, R. White, E. Bañados, E. F. Bell, R. Bender, E. J. Bernard, M. Boegner, F. Boffi, M. T. Botticella, A. Calamida, S. Casertano, W.-P. Chen, X. Chen, S. Cole, N. Deacon, C. Frenk, A. Fitzsimmons, S. Gezari, V. Gibbs, C. Goessl, T. Goggia, R. Gourgue, B. Goldman, P. Grant, E. K. Grebel, N. C. Hambly, G. Hasinger, A. F. Heavens, T. M. Heckman, R. Henderson, T. Henning, M. Holman, U. Hopp, W.-H. Ip, S. Isani, M. Jackson, C. D. Keyes, A. M. Koekemoer, R. Kotak, D. Le, D. Liska, K. S. Long, J. R. Lucey, M. Liu, N. F. Martin, G. Masci, B. McLean, E. Mindel, P. Misra, E. Morganson, D. N. A. Murphy, A. Obaika, G. Narayan, M. A. Nieto-Santisteban, P. Norberg, J. A. Peacock, E. A. Pier, M. Postman, N. Primak, C. Rae, A. Rai, A. Riess, A. Riffeser, H. W. Rix, S. Röser, R. Russel, L. Rutz, E. Schilbach, A. S. B. Schultz, D. Scolnic, L. Strolger, A. Szalay, S. Seitz, E. Small, K. W. Smith, D. R. Soderblom, P. Taylor, R. Thomson, A. N. Taylor, A. R. Thakar, J. Thiel, D. Thilker, D. Unger, Y. Urata, J. Valenti, J. Wagner, T. Walder, F. Walter, S. P. Watters, S. Werner, W. M. Wood-Vasey, R. Wyse, The Pan-STARRS1 Surveys. arXiv:1612.05560 (2016).

191. A. Mainzer, J. Bauer, T. Grav, J. Masiero, R. M. Cutri, J. Dailey, P. Eisenhardt, R. S. McMillan, E. Wright, R. Walker, R. Jedicke, T. Spahr, D. Tholen, R. Alles, R. Beck, H. Brandenburg, T. Conrow, T. Evans, J. Fowler, T. Jarrett, K. Marsh, F. Masci, H. McCallon, S. Wheelock, M. Wittman, P. Wyatt, E. De Baun, G. Elliott, D. Elsbury, T. Gautier IV, S. Gomillion, D. Leisawitz, C. Maleszewski, M. Micheli, A. Wilkins, Preliminary results from NEOWISE: An enhancement to the wide-field infrared survey explorer for solar system science. *Astrophys. J.* **731**, 53 (2011).

192. NEOWISE, NEOWISE-R Single Exposure (L1b) Source Table (2020); https://catcopy.ipac.caltech.edu/dois/doi.php?id=10.26131/IRSA144.

193. J. L. Tonry, L. Denneau, H. Flewelling, A. N. Heinze, C. A. Onken, S. J. Smartt, B. Stalder, H. J. Weiland, C. Wolf, The ATLAS all-sky stellar reference catalog. *Astrophys. J.* **867**, 105 (2018).

194. P. W. A. Roming, T. E. Kennedy, K. O. Mason, J. A. Nousek, L. Ahr, R. E. Bingham, P. S. Broos, M. J. Carter, B. K. Hancock, H. E. Huckle, S. D. Hunsberger, H. Kawakami, R. Killough, T. S. Koch, M. K. Mclelland, K. Smith, P. J. Smith, J. C. Soto, P. T. Boyd, A. A. Breeveld, S. T. Holland, M. Ivanushkina, M. S. Pryzby, M. D. Still, J. Stock, The swift ultra-violet/optical telescope. *Space Sci. Rev.* **120**, 95–142 (2005).

195. D. N. Burrows, J. E. Hill, J. A. Nousek, J. A. Kennea, A. Wells, J. P. Osborne, A. F. Abbey, A. Beardmore, K. Mukerjee, A. D. T. Short, G. Chincarini, S. Campana, O. Citterio, A. Moretti, C. Pagani, G. Tagliaferri, P. Giommi, M. Capalbi, F. Tamburelli, L. Angelini, G. Cusumano, H. W. Bräuninger, W. Burkert, G. D. Hartner, The swift X-ray telescope. *Space Sci. Rev.* **120**, 165–195 (2005).

196. M. Riello, F. De Angeli, D. W. Evans, P. Montegriffo, J. M. Carrasco, G. Busso, L. Palaversa, P. W. Burgess, C. Diener, M. Davidson, N. Rowell, C. Fabricius, C. Jordi, M. Bellazzini, E. Pancino, D. L. Harrison, C. Cacciari, F. van Leeuwen, N. C. Hambly, S. T. Hodgkin, P. J. Osborne, G. Altavilla, M. A. Barstow, A. G. A. Brown, M. Castellani, S. Cowell, F. De Luise, G. Gilmore, G. Giuffrida, S. Hidalgo, G. Holland, S. Marinoni, C. Pagani, A. M. Piersimoni, L. Pulone, S. Ragaini, M. Rainer, P. J. Richards, N. Sanna, N. A. Walton, M. Weiler, A. Yoldas, Gaia early data release 3. Photometric content and validation. *Astron. Astrophys.* **649**, A3 (2021).

197. R. C. Kennicutt Jr., Star formation in galaxies along the hubble sequence. *Ann. Rev. Astron. Astrophys.* **36**, 189–232 (1998).

198. L. J. Kewley, M. J. Geller, R. A. Jansen, [O II] as a star formation rate indicator. *Astron. J.* **127**, 2002–2030 (2004).

199. Y. Zu, C. S. Kochanek, B. M. Peterson, An alternative approach to measuring reverberation lags in active galactic nuclei. *Astrophys. J.* **735**, 80 (2011).

200. Y. Zu, C. S. Kochanek, S. Kozłowski, A. Udalski, Is quasar optical variability a damped random walk? *Astrophys. J.* **765**, 106 (2013).

201. N. Jiang, T. Wang, L. Dou, X. Shu, X. Hu, H. Liu, Y. Wang, L. Yan, Z. Sheng, C. Yang, L. Sun, H. Zhou, Mid-infrared outbursts in nearby galaxies (MIRONG). I. Sample selection and characterization. *Astrophys. J. Suppl. Ser.* **252**, 32 (2021).
